# Identification of selective inhibitors of *Helicobacter pylori* IMPDH as a targeted therapy for the infection

**DOI:** 10.1038/s41598-018-37490-x

**Published:** 2019-01-17

**Authors:** Kapil Juvale, Gayathri Purushothaman, Vijay Singh, Althaf Shaik, Srimadhavi Ravi, Vijay Thiruvenkatam, Sivapriya Kirubakaran

**Affiliations:** 1 Chemistry, Indian Institute of Technology Gandhinagar, Palaj, Gandhinagar, 382355 India; 2 Biological engineering, Indian Institute of Technology Gandhinagar, Palaj, Gandhinagar, 382355 India; 3 Physics, Indian Institute of Technology Gandhinagar, Palaj, Gandhinagar, 382355 India; 4 Present Address: Shobhaben Pratapbhai Patel School of Pharmacy & Technology Management, SVKM’s NMIMS, V.L. Mehta Road, Vile Parle (W), Mumbai, 400056 India

## Abstract

*Helicobacter pylori* (*H. pylori*), the major cause of several gastric disorders has been recognied as a type I carcinogen. By virtue of resistance developed by *H. pylori* strains, currently used antibiotic based treatments rather demonstrate high failure rates. Hence, there is an emerging need for identification of new targets to treat *H. pylori* infection. Inosine-5′-monophosphate dehydrogenase (IMPDH) has been studied as a potential target to treat *H. pylori* infection. Here, a detailed enzyme kinetic study of recombinant expressed *H. pylori* inosine-5′-monophosphate dehydrogenase (*Hp*IMPDH) is presented. A new in-house synthesized indole-based scaffold is identified as an inhibitor for *Hp*IMPDH. These indole-based compounds showed non-competitive inhibition against IMP and NAD^+^ whereas the benzimidazole compounds were found be uncompetitive inhibitors. The new indole scaffold ensures specificity due to its high selectivity for bacterial IMPDH over human IMPDH II. Our work aims to overcome the drawback of existing inhibitors by introducing new indole scaffold for targeting bacterial IMPDH.

## Introduction

Worldwide more than 50% population is infected by *Helicobacter pylori* (*H. pylori*), a gram-negative bacterium which resides in the antral region of stomach^1^. The infection rate is much higher (up to 80%) in developing countries. Due to the lack of hygiene and sanitation, in India alone more than 20 million people are estimated to be suffering from gastric ulcer^2^. Although *H. pylori* do not show symptoms in most of the population, it has been associated with various disorders including gastric and duodenal ulcers, gastric atrophy and gastritis^3^. *H. pylori* is categorized as type I carcinogen due to its involvement in the pathology of gastric adenocarcinoma and malt associated lymphoid tissue lymphoma (MALT lymphoma)^4^. Currently, *H. pylori* infection is treated by either first line clarithromycin-based triple therapy or a quadruple therapy replacing clarithromycin by new antibiotics^5^. Despite these efforts, there is an increase in the antibiotic resistance shown by various strains of *H. pylori*. Hence, there is an emerging need to identify new targets to treat *H. pylori* infection, especially in developing countries like India.

Inosine-5′-monophosphate dehydrogenase (IMPDH, guaB, EC:1.1.1.205) has been demonstrated to be a promising target for several diseases. IMPDH is reported as a potential target for treating some pathogens like *Cryptosporidium parvum* (*C. parvum*) and *Mycobacterium tuberculosis* (*M. tuberculosis*)^6,7^. In the de novo biosynthesis of purine nucleotides, IMPDH enzyme is responsible for the oxidation of inosine 5′-monophosphate (IMP) to xanthosine 5′-monophosphate (XMP). The XMP is then further converted into guanosine 5′-monophosphate (GMP) by GMP synthase. Successive actions of several enzymes on GMP gives rise to some of the building blocks of DNA (dGTP) and RNA (dGTP)^8,9^. Hence, inhibition of IMPDH can stop the expansion of the guanine nucleotide pool that is needed for the microbial proliferation.

The phylogenetic analysis of *H. pylori* IMPDH (*Hp*IMPDH) revealed its close resemblance to the *C. parvum* IMPDH (*Cp*IMPDH) with a sequence identity of 60%. This fact justifies the investigation of already available bacterial IMPDH inhibitors for *Hp*IMPDH. Moreover, this similarity enables the designing of new inhibitors by *in silico* docking studies. Due to fundamental differences in the structural and kinetic properties of prokaryotic IMPDH (Pro-IMPDHs) and eukaryotic IMPDH (Euk-IMPDHs), it is possible to develop selective inhibitors of bacterial enzyme^10^.

Gollapalli *et al*. have reported several inhibitors of *Cp*IMPDH having selectivity over host (human) IMPDH II. It was shown that the benzimidazole class of small molecules are the most promising inhibitors of bacterial IMPDH. These compounds were found to bind in the cofactor (NAD^+^) binding pocket with one of the aromatic rings having π-π interactions with IMP^6,11,12^. Even in eukaryotes, the IMP binding site of bacterial IMPDH enzyme is highly conserved. Hence, inhibitors binding to the IMP binding site can also inhibit the host (human) IMPDH and lead to toxicity. Therefore, a competitive inhibition with IMP is the least desirable, necessitating identification of mechanism for bacterial IMPDH inhibition.

**Figure 1:**
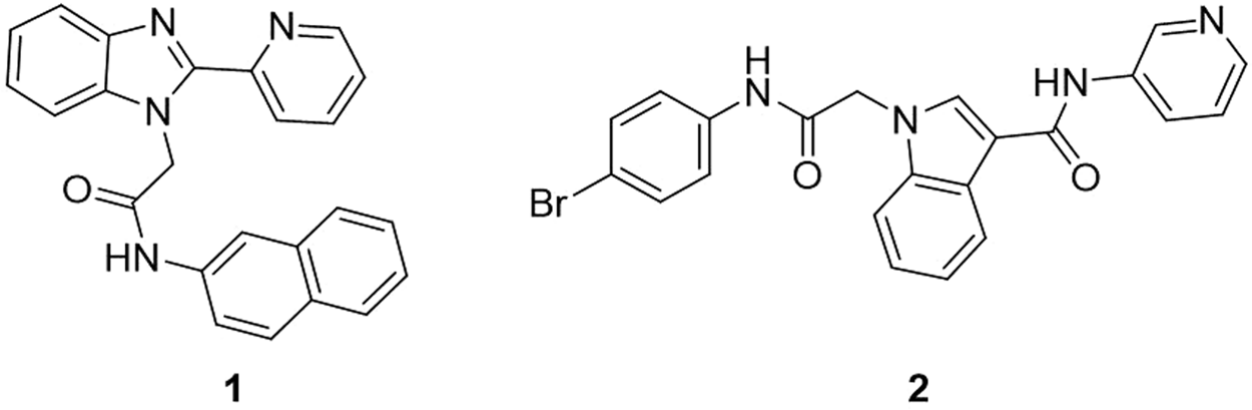
Structures of reported benzimidazole (**1**,**C91**) and synthesised indole (**2**) based small molecules selected for the study.

Although C91 (**1**), a benzimidazole based inhibitor is one of the most potent *Hp*IMPDH inhibitor known, the benzimidazole scaffold suffers from limitations due to its poor metabolic profile^13^. With the aim of identifying new selective inhibitors of this bacterial IMPDH, we synthesized several indole-based small molecules. Indole based molecules have previously been identified as inhibitors of human IMPDH but with different structural profile^14^. The molecules studied here were designed to avoid any inhibition of the host IMPDH. These molecules were investigated for their inhibitory potential against *Hp*IMPDH and *Homo sapiens* IMPDH (*Hs*IMPDH). From the inhibitory studies, we found that these small molecule inhibitors have a good inhibitory potential with selectivity over host IMPDH. Further, we have taken efforts to study the mechanism of *Hp*IMPDH inhibition with selected benzimidazole and indole-based small molecules (Fig. 1).

We found that the known *Hp*IMPDH inhibitor **1**^6^, a benzimidazole-based small molecule, is an uncompetitive IMPDH inhibitor with respect to both the substrates IMP and NAD^+^. Interestingly the indole-based small molecule **2** that are carefully designed and synthesized found to be noncompetitive inhibition towards *Hp*IMPDH versus both IMP and NAD^+^.

These findings indicated that the novel indole-based compound binds to different complexes of IMPDH and its substrates. This would be beneficial to design new and selective inhibitors of *Hp*IMPDH, opening doors for developing highly selective inhibitors of *Hp*IMPDH. Since competitive IMPDH inhibitors with respect to IMP binding site lack selectivity over host, uncompetitive and non-competitive inhibitors are much preferable. Notably in case of such inhibitors, increase in the substrate concentration does not affect the affinity of the inhibitors^15^.

## Results and Discussion

### Cloning, Expression, Purification and Characterization of *Hp*IMPDH

**Figure 2:**
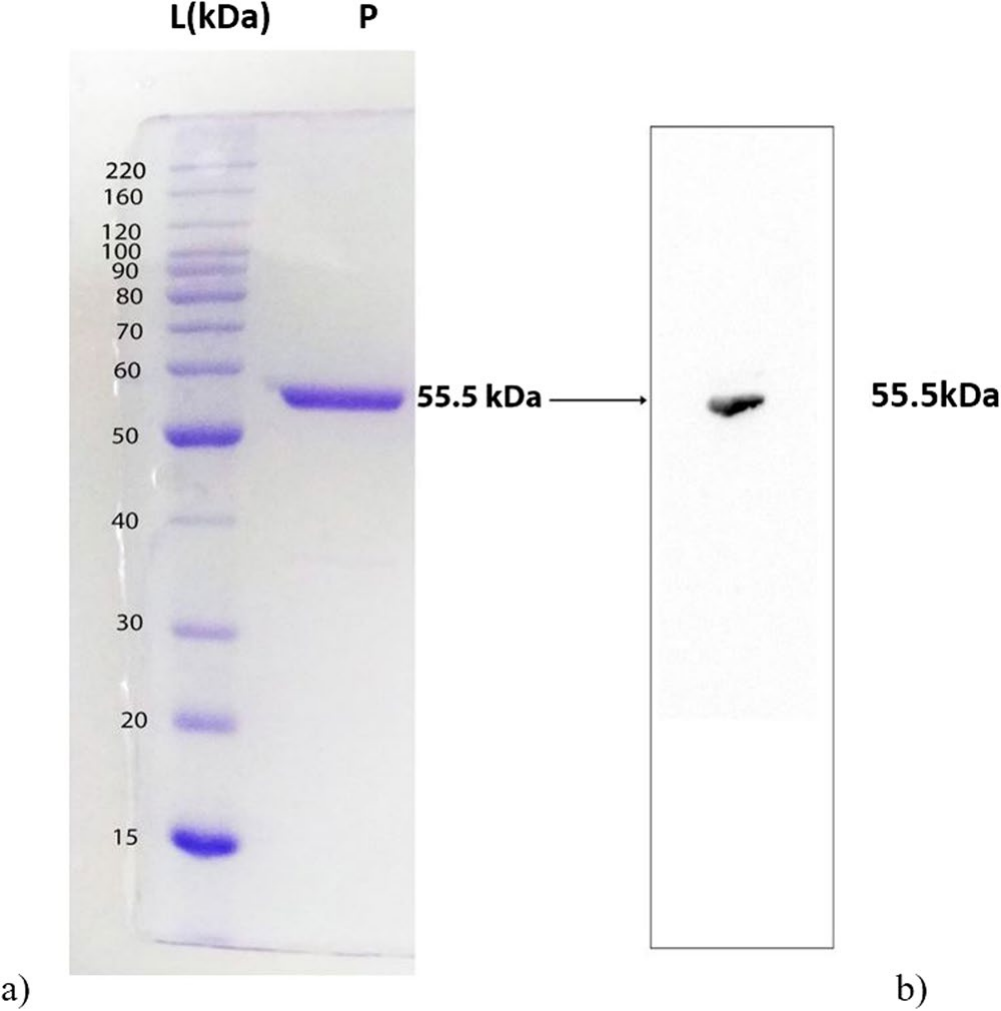
(**a**) SDS-PAGE gel; L-Ladder and P-Purified *Hp*IMPDH protein (**b**) Western blot result of purified recombinant *Hp*IMPDH. Full-length blots/gels are presented in Supplementary Fig. S15.

The recombinant *Hp*IMPDH was expressed in Rosetta™(DE3) pLysS using pET-28a(+) expression vector. The protein was purified using affinity chromatography (Ni-NTA) and size exclusion chromatography. Further, the recombinant *Hp*IMPDH was confirmed by western blot using anti His-tag antibody (Fig. 2). The final concentration of the protein was found to be 3 mg/mL (total volume of 1 mL) from one-liter culture. The characterisation of recombinant *Hp*IMPDH by LC-MS/MS resulted in 65% sequence match using peptide mass fingerprint analysis between recombinant *Hp*IMPDH and the *Hp*IMPDH protein sequence obtained from the database.

**Figure 3:**
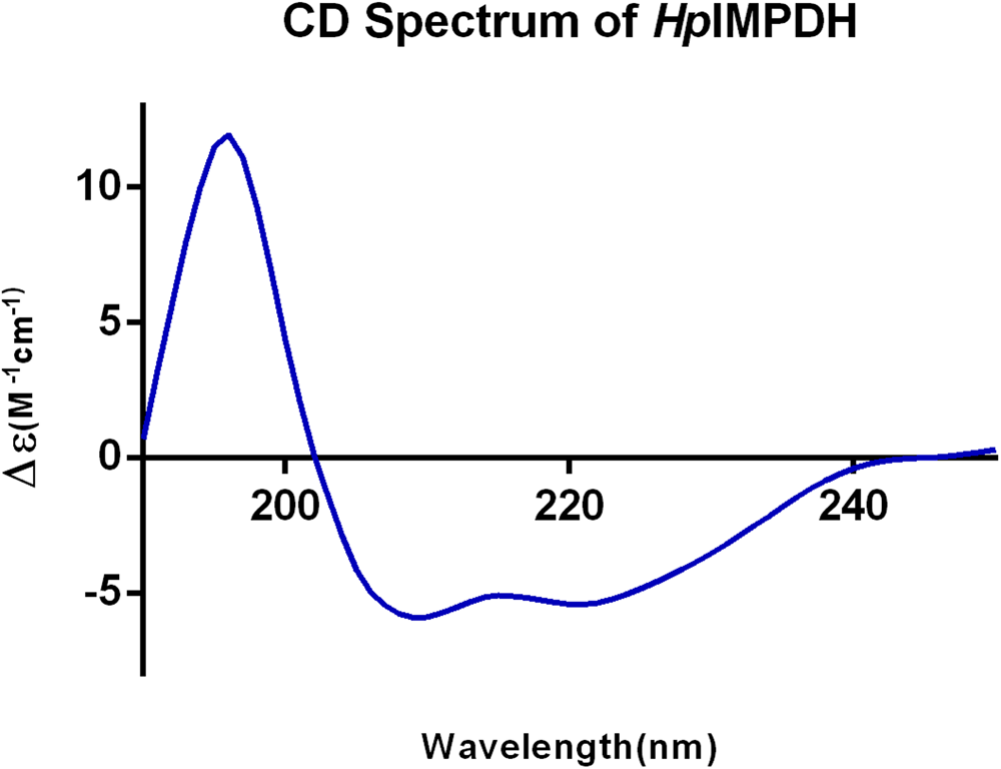
CD spectrum of purified *Hp*IMPDH indicating the presence of alpha helix (negative peak at 208 and 222 nm) and beta strand (positive peak at 196 nm).

The secondary structure prediction of purified recombinant *Hp*IMPDH is analyzed via Circular Dichroism (CD) (Fig. 3). A negative peak at 208 nm and 222 nm in the spectra indicated presence of alpha helices and a positive peak at 196 nm suggested presence of beta sheets^16,17^. The quantification of the secondary structure elements^18,19^ showed that *Hp*IMPDH comprises of 37% alpha helices, 17% of beta strand, 7% turns and 21% others(loops). The *Hp*IMPDH secondary structure results obtained from CD were compared with the secondary structures of other Pro-IMPDHs and Euk-IMPDHs (Table S5). The reported CD spectra^20^ and crystal structure of other IMPDHs showed the presence of 22–34% alpha helices and 9–29% of beta sheets^21^. Hence, this confirms the secondary structure of *Hp*IMPDH found to be in correlation with the other reported IMPDHs.

### Enzymatic activity and kinetics

**Figure 4:**
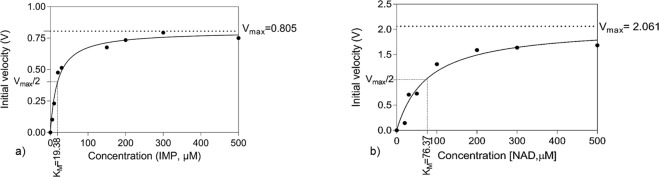
Enzyme activity of *Hp*IMPDH was determined in the presence of (**a**) Varying concentrations of IMP and (**b**) Varying concentrations of NAD^+^. The curves were fitted using the Michaelis-Menten equation.

Before carrying out enzymatic assays, it is important to determine the enzyme activity. After confirming the activity, the recombinant *Hp*IMPDH (100 nM) was utilized for the enzyme kinetic studies. The studies were performed by varying IMP concentrations while keeping NAD^+^ concentration (600 µM) constant and by varying NAD^+^ concentration while keeping IMP concentration (500 µM) constant to study the kinetics of *Hp*IMPDH for substrate (IMP) as well as for cofactor (NAD^+^). As reported earlier by Gollapalli *et al*., *Hp*IMPDH follows the Michaelis-Menten type of kinetics for both IMP and NAD^+^, the plots of initial velocity versus NADH concentration were found to be hyperbolic^6^ (Fig. 4). The K_M_ values of *Hp*IMPDH for IMP and NAD^+^ were found to be 19.38 ± 1.53 µM and 76.37 ± 1.09 µM respectively. In addition, the k_cat_ values of the enzyme for the substrates IMP and NAD^+^ were found to be 5.57 ± 0.88 (S^−1^) and 5.96 ± 0.53 (S^−1^) respectively. The kinetics of *Hp*IMPDH was then compared with other Pro-IMPDHs and Euk-IMPDHs (Table S6), which showed the broad differences of values among them. The K_M_ (IMP) of *Hp*IMPDH was comparable with the *Cp*IMPDH and *Bp*IMPDH (Pro-IMPDH).

### Enzyme inhibition and mechanistic studies

**Figure 5:**
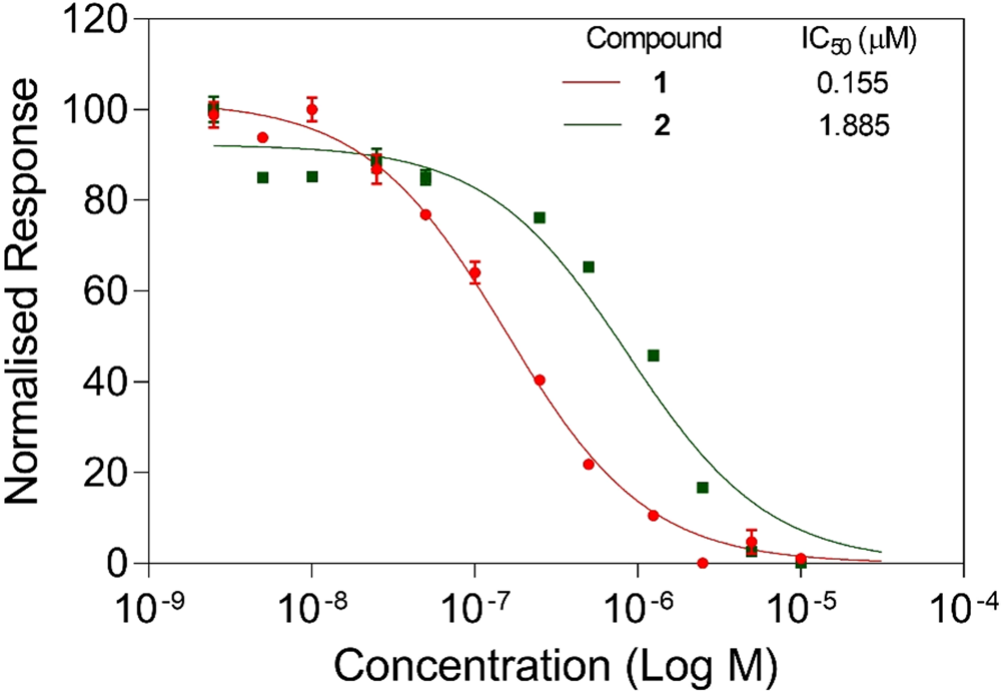
Concentration response curve for the inhibitory effect of **1 (C91)** and **2** against *Hp*IMPDH. The data were fitted using 4 parameter Hill equation and each data point represents mean of triplicate.

The inhibitory effect of the newly synthesised indole-based molecule (**2**) and selected benzimidazole small molecule (**1**) against *Hp*IMPDH is investigated by monitoring the change in the initial velocity of NADH formation. To calculate the IC_50_ values, the initial velocity of the reaction is plotted against concentrations of the small molecule inhibitor, and the data is fitted with the Hill equation. A representative concentration-response curve for compound **1** and **2** are shown in Fig. 5.

The IC_50_ value of selected benzimidazole and indole-based small molecules are given in Tables 1 and S3. The indole-based molecules when screened at 10 μM concentration showed no substantial effect on the enzyme activity of human IMPDH-II (Fig. S8). This suggests the selectivity of the indole-based molecules towards the *Hp*IMPDH.

**Table 1:** *Hp*IMPDH inhibition data for compounds **1** and **2**.

Compounds	IC_50_(µM)
1	0.155 ± 0.082
2	1.885 ± 0.42

There are three important mechanisms of enzyme inhibition, viz. competitive, non-competitive and uncompetitive inhibition. The IMP binding site is highly conserved in all the IMPDHs. Hence, any inhibitor that shows competitive inhibition against IMP would lose selectivity over host IMPDH and subsequently would lead to undesirable side effects. Therefore, to find out the type of inhibition by the selected small molecule inhibitors, kinetic studies were performed in the presence of several concentrations of the inhibitor and varying concentration of IMP or NAD^+^. The initial velocity data were plotted using Lineweaver-Burk equation using GraphPad Prism.

**Figure 6:**
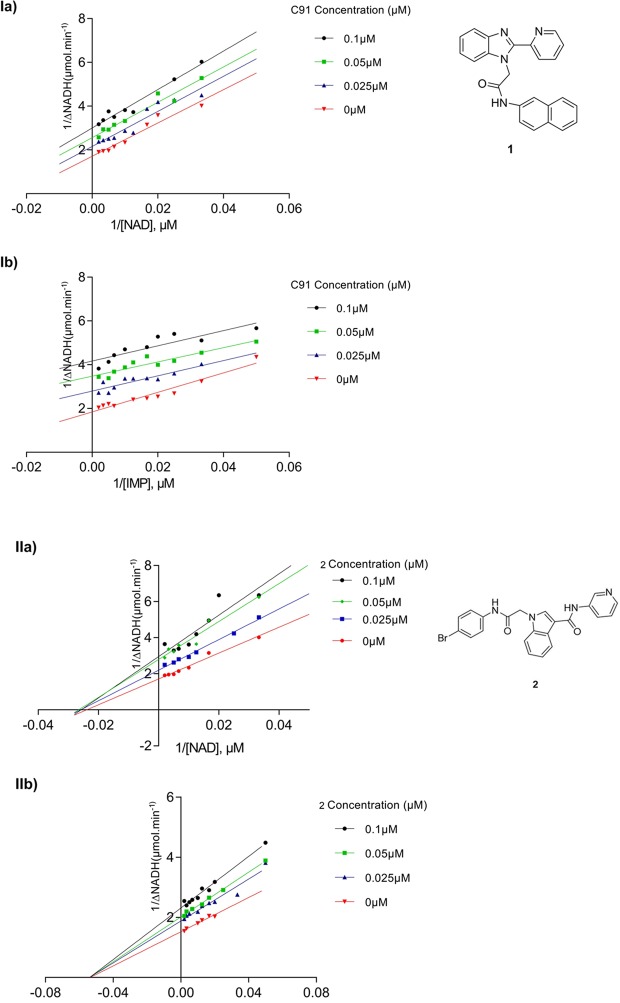
Mechanism of *Hp*IMPDH inhibition by small molecules **1** (**C91**) and **2** was studied against varying concentrations of IMP and NAD^+^. The data is plotted using the Lineweaver-Burk equation.

As in Fig. 6: Ia and Ib, compound **1** inhibited *Hp*IMPDH in an un-competitive manner against both IMP and NAD^+^. The K_M_ and V_max_ both found to be reduced with an increase in the inhibitor concentration, suggesting binding of the inhibitor to the substrate-enzyme complex. This important finding stresses the selectivity of the molecule towards bacterial IMPDH over human IMPDH. In contrast, IMP analogues have previously been shown to be competitive inhibitors against IMP and noncompetitive inhibitors against NAD^+^. The uncompetitive behaviour of **1** towards both IMP and NAD^+^ suggests that it has a strong preference for binding to the E-XMP* complex^15^. This will be highly advantageous *in-vivo* as increased substrate concentration would lead to increased inhibition of the IMPDH unlike competitive behavior. First generation C series inhibitors have been found to be the non-competitive inhibitors^6^, whereas the second-generation **1** molecule^12^ showed uncompetitive inhibition. Mycophenolic acid (MPA), one of the potent inhibitor of *Hs*IMPDH (both I and II) has also shown a similar inhibition mechanism against both IMP and NAD^+^ ^22^.

In the current study, we have also investigated new indole-based molecules for their ability to inhibit *Hp*IMPDH (inhibition data given in supplementary information). In the inhibitory studies, it was observed that compound **2** was able to inhibit *Hp*IMPDH with an IC_50_ value in the micromolar range. The kinetic studies carried out with compound **2** showed inhibitory behaviour different from that of the **C91**. As can be seen from Fig. 6-IIa and IIb, compound **2** showed a typical Lineweaver Burk plot for non-competitive inhibition against both IMP and NAD^+^, where regression lines meet on the X-axis. Here, the K_M_ values of the *Hp*IMPDH remained constant since the inhibitor does not bind to the active site. In addition, there was a decrease in the V_max_ with the increasing concentration of inhibitor due to the low functional enzyme concentration.

In the case of non-competitive inhibition, the inhibitor binds to an allosteric site and changes the conformation of the active site. This type of inhibition is independent of substrate concentration as the inhibitor has the ability to bind and inhibit the free enzyme as well as the enzyme-substrate complex (unlike uncompetitive inhibitors that can inhibit only enzyme-substrate complexes). This suggests a preference of the inhibitor molecule **2** for the E•IMP and E-XMP* complexes, making the enzyme incapable of catalysing IMP to XMP conversion due to conformational changes in the IMP and NAD^+^ binding sites. The same kind of mechanism has been observed for known IMPDH inhibitors TAD (thiazole-4-carboxamide adenine dinucleotide) and SAD (selenium analogue)^23^.

### Multiple Sequence Alignment (MSA) of *Hp*IMPDH with IMPDHs of other organisms

**Figure 7:**
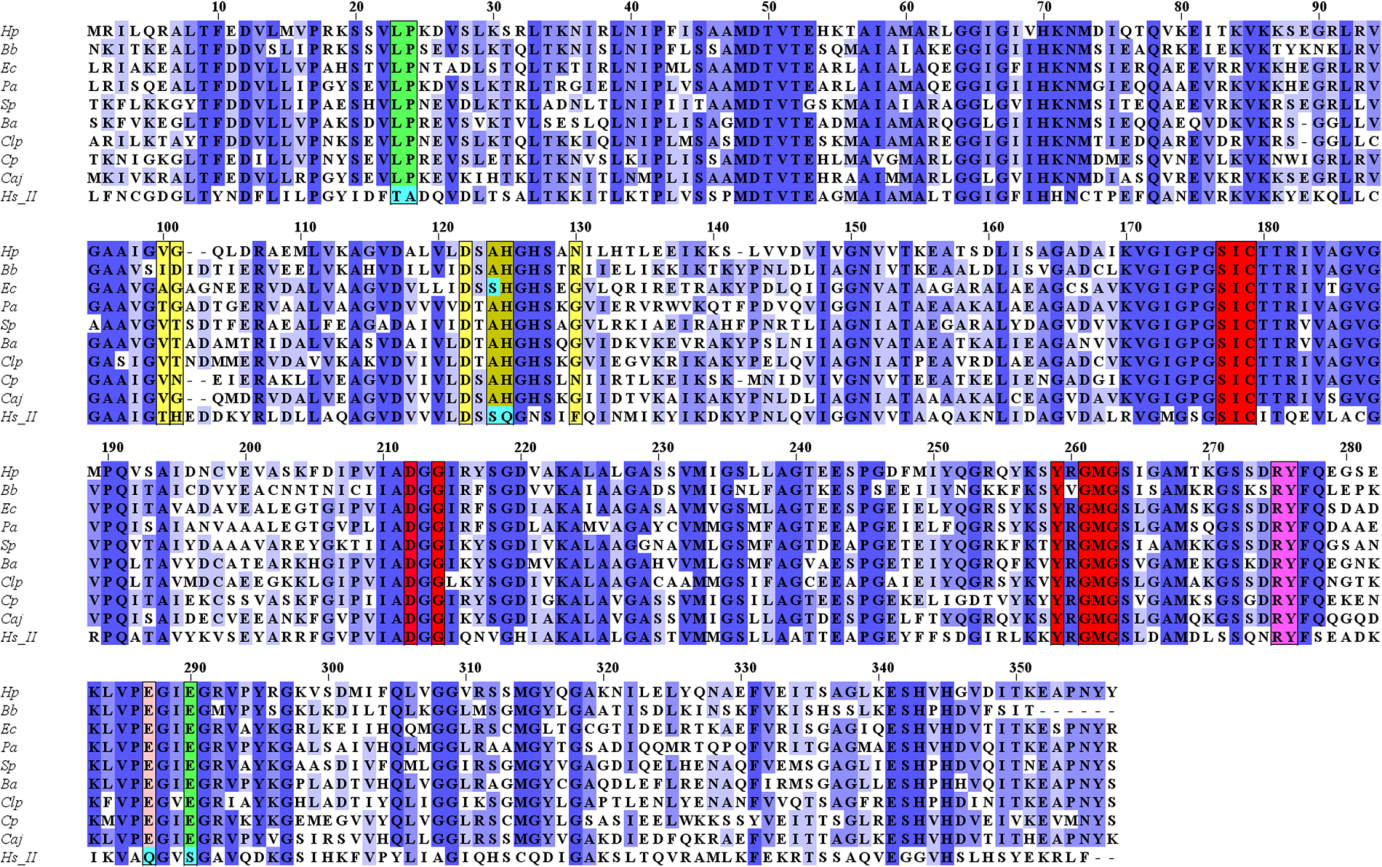
Multiple sequence alignment of various Pro-IMPDHs including *Hp*IMPDH with *Hs*IMPDH-II: Intensity of the purple colour indicates the percentage of the sequence conservation; Residues binding to C91 are coloured in green (*Hp*, *Bb*, *Pa*, *Sp.Ba*, *Clp*, *Cp*, *Caj*) and cyan(*Ec*, *Hs*_*II*); Residues interacting with NAD^+^ in *Hs*IMPDH-II and the corresponding residues in the Pro-IMPDHs are in indicated in yellow; Residues interacting with both 1 and NAD^+^ are noted in greenish yellow; Residues interacting with IMP are indicated in red; Conserved residues RY in the flap region is indicated in the magenta; Key residue binding to the Mycophenolic acid is coloured in cyan for *Hs*IMPDH-II and the corresponding residues in the Pro-IMPDHs coloured in peach.

MSA is performed to identify the suitable drug-binding site in the bacterial IMPDH enzyme that affects specificity over host IMPDH. MSA of *Hp*IMPDH with other Pro-IMPDHs and *Hs*IMPDH-II is carried out. The protein sequences of *Hp*IMPDH taken for analysis is carfully studied with the reported crystal structures of IMPDH for designing small molecules. The result of MSA gave a clear assessment of conserved and non-conserved residues in *Hp*IMPDH as compared to other IMPDHs. The residues involved in key interactions are (Fig. 7), are conserved among all IMPDHs^15,24^. The Fig. 7 shows the conserved residues among the Pro-IMPDHs with respect to *Hp*IMPDH and the *Hs*IMPDH-II. The intensity of the purple colour corresponds to the percentage conservation of the residues among the species. Among the Pro-IMPDHs, *Hp*IMPDH has a higher percentage of sequence similarity with *Campylobacter jejuni* IMPDH (*Cj*IMPDH) and *Cp*IMPDH.

The sequence comparison of *Hp*IMPDH with Euk-IMPDHs has revealed that the key binding residue of Mycophenolic acid (*Hs*IMPDH-II inhibitor)^25^ is different in Euk-IMPDHs (Gln400) and *Hp*IMPDH (Glu419). However, these residues are conserved in their own class. Reported crystal structures of *Bacillus anthracis* IMPDH (*BaI*MPDH) [PDB ID: 4MY9], *Clostridium perfringens* (*Clp*IMPDH) [PDB ID: 4Q33] and *Cj*IMPDH [PDB IDL 4MZ8] with known inhibitor **1**^26–28^ suggest an interaction of the compound with Glu290 by a hydrogen bond and appears to be a key interaction between Pro-IMPDH and **1**. This Glu290 residue is conserved over Pro-IMPDH but it is replaced by Ser290 in *Hs*IMPDH-II suggesting species-specific inhibitor of IMPDH (Fig. 7). Other residues reported to be involved in interaction with **1** via hydrophobic interactions are Leu23, Pro24, Ala125, His126, Met268. Except Met268 other residues are not conserved in Pro-IMPDHs and *Hs*IMPDH-II. **1** effectively interacts only with two of the amino acid residues (Ala125 and His126) that bind to NAD^+^ through hydrophobic interactions. Leu23, Pro24, Met268and Glu290 are other amino acids which are not a part of either IMP or NAD^+^ binding site.

MSA results suggested that **1** is not a competitive inhibitor and these observations agreed with our experimental results. The residues, 280–340 (Fig. 7), form a part of the flap region of the active site binds to NAD^+^. This region is highly divergent in *Hs*IMPDH-II and Pro-IMPDHs which make this region a suitable target to design species-specific inhibitors^6,15,29,30^.

### Prediction of the binding site

**Figure 8:**
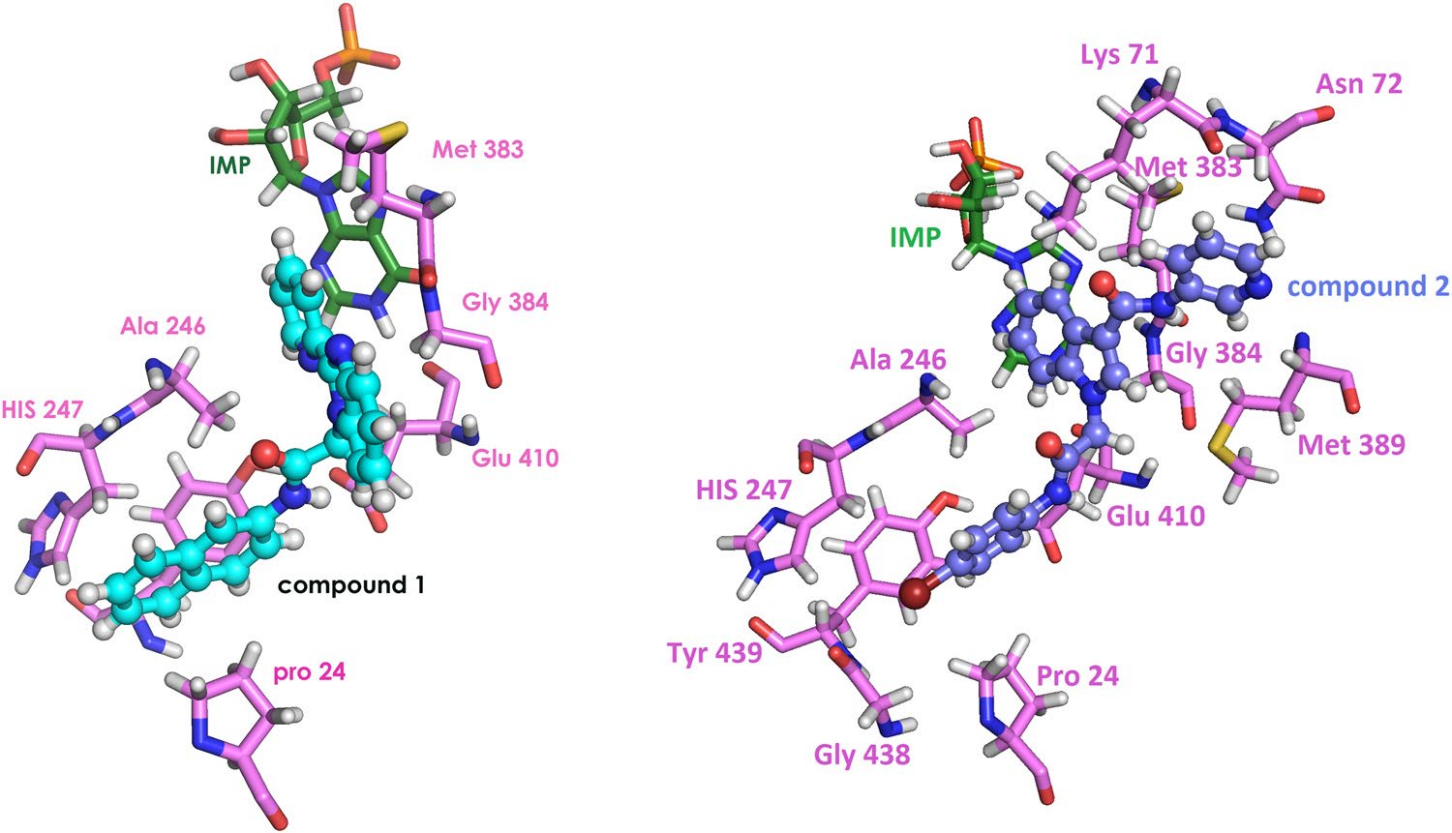
Interactions of compounds **1** and **2** at the binding site of *Hp*IMPDH.

To analyse the binding site of the inhibitors in the protein, the 3D- structure of *Hp*IMPDH has been generated from *S. Pyorgenes* (PDB ID: 1ZFJ) by SWISS-MODEL^31^ and validated. The generated structure was utilised to examine the mode of binding of non-competitive and uncompetitive inhibitors to the *Hp*IMPDH in an attempt to rationalise its activity against *Hp*IMPDH. **1** was found to be binding to the protein with the best ranking and docking score of –5.3 kcal mol^−1^. The binding mode of **1** is similar to the way **C64** (C series *Cp*IMPDH inhibitor) binds to the *Cp*IMPDH^6^. The naphthyl ring of **1** was found to be involved in π-π interaction with His 247  and Pro 24. The pyridyl ring forms a hydrophobic interaction with hypoxanthine ring of IMP and Met 383 (Fig. 8).

**2** (non-competitive inhibitor) was found to be binding to the protein with the docking score of –6.9 kcal mol^−1^. The 4-bromo phenyl group of the compound was found to be stabilized by hydrophobic interactions with residues His 247, Tyr 439, Gly 438 and Pro 24. Similar binding was reported in the co-crystal of *Tf*IMPDH with thiazole-4-carboxamide adenine dinucleotide (TAD)^32^. Further, **2** was stabilized by hydrogen bonding with Lys 71 and Met 383. In **2** the N-phenyl-1H-indole-3-carboxamide moiety makes different interaction with IMPDH and orients into top right hydrophobic region. These strong interactions of **2** at the active site can be reasoned for its activity towards *Hp*IMPDH (Fig. 8)

## Conclusion

Bacterial IMPDH, a vital enzyme in the nucleotide biosynthesis pathway has recently received a lot of attention as a possible target to treat multi-drug resistant infections. In the current work, the recombinant *Hp*IMPDH kinetic profile was characterized and it showed kinetic behavior of bacterial IMPDH and distinct from that of *Hs*IMPDH-I and II. These results suggest the possibility of selective inhibition of the *Hp*IMPDH. We are reporting a new indole-based scaffold to target the *Hp*IMPDH that shows a good inhibition against *Hp*IMPDH and not against HsIMPDH-II confirming its selectivity over the host IMPDH. Benzimidazole based molecules have limited use due to their bad metabolite profile that could be overcome with development of indole based small molecule inhibitors of bacterial IMPDH.

In the current study, we have also studied the mechanism of inhibition of selected inhibitors for both the substrates, IMP and NAD^+^. From the results, it is clearly observed that the structural changes in the inhibitor influences not only the effectiveness (IC_50_) of the inhibitor but also affects the affinity of the enzyme/enzyme-substrate/enzyme-product complexes. A well-known inhibitor **C91** bearing a benzimidazole scaffold showed an uncompetitive behavior against IMP as well as NAD^+^ while newly synthesized indole-based compound (**2**) showed a noncompetitive inhibition. Both benzimidazole and indole-based inhibitors have the advantage that the inhibitory effect of these compounds will not be affected by the increase in the substrate concentration. This is in contrast to the competitive inhibitors, where bacteria can gain the resistance by producing the substrates in higher concentrations to overcome the effect of inhibitors. The findings from the current study are encouraging as we were able to identify new indole-based molecules for the IMPDH inhibition that could be the new starting point towards the quest of finding better bacterial IMPDH inhibitors to fight infections.

## Experimental Section

### Identification of *Hp*IMPDH by Mass spectrometry – Peptide mapping

LC-MS/MS peptide mapping analysis was performed to confirm the expression of *Hp*IMPDH in *E. coli*. The purified protein sample was excised from the gel and tryptic digestion carried out for the LC-MS/MS analysis (Proteomics facility, MBU, IISc, Bangalore). The resultant data were examined for Peptide Mass Fingerprinting (PMF) using MASCOT software. The MASCOT search matched digested peptide sequence with the *Hp*IMPDH protein sequences available in the protein database (NCBI and SwissProt). Validation of protein identification was done based on the number of matched peptides, the extent of protein sequence coverage and the score probability^33^ (Tables S1 and S2).

### Secondary structure characterization by CD spectroscopy

To analyze the secondary structure content, recombinant *Hp*IMPDH protein (700 µg/ml) in 20 mM Tris-Cl and 50 mM NaCl buffer (pH 7.8) was used for CD spectra analysis. The analysis was carried out using Jasco spectropolarimeter equipped with a peltier-type temperature control system at a wavelength scan of 190 nm to 250 nm. Far UV CD spectra were recorded in 0.5 mm quartz cuvette at 25 °C. A signal-averaged over at least 3 scans were collected. The spectrum was analyzed for secondary structure content using CDSSTR software^18^ which was included in Dichroweb tool^19^.

### Enzymatic activity and steady state kinetics of *Hp*IMPDH

IMPDH enzyme catalyzes the oxidation of IMP to XMP with concurrent reduction of NAD^+^ to NADH. The formation of NADH can be monitored by measuring absorbance at 340 nm or fluorescence at 440 nm when excited at 340 nm. In presence of active IMPDH enzyme, there is an increase in the formation of NADH which could be correlated with the catalytic activity of the enzyme.

Kinetic studies of recombinant *Hp*IMPDH were performed with an assay buffer containing 50 mM Tris-Cl pH 8, 100 mM KCl and 1 mM dithiothreitol (DTT) at a final volume of 200 μL using black96F well plate (Tarsons Products Pvt. Ltd., Kolkata, India). Assay was performed using 100 nM *Hp*IMPDH in the presence of varying concentrations of IMP or NAD^+^. The production of NADH was monitored by measuring fluorescence at 440 nm when excited at 340 nm using PerkinElmer EnVision Multilabel Reader (Perkin Elmer, Inc., MA, USA).

For determination of Michaelis–Menten constant (K_M_), data of initial velocity at varying concentrations of IMP (5 µM to 500 µM) at fixed NAD^+^ concentration (600 µM) or varying concentrations of NAD^+^ (10 µM to 500 µM) at fixed IMP concentration (500 µM) were determined. The initial velocity data was fitted using Michaelis–Menten equation (1) with the help of GraphPad Prism to calculate enzyme kinetic parameters.

1 $${\rm{V}}=\frac{{\rm{V}}\,{\rm{\max }}\,[{\rm{S}}]}{{\rm{KM}}+[{\rm{S}}]}$$

where, V is velocity, V_max_ is maximum velocity, [S] is substrate concentration, K_M_ is Michaelis–Menten constant.

### Enzyme inhibition assay

The inhibitory effects of the benzimidazole and indole-based small molecules are listed in Fig. 1. Similar to enzyme kinetic studies, these were also determined by monitoring NADH concentration through the fluorescence measurement. In the presence of IMPDH inhibitor, decreased catalytic activity of the enzyme reduces the formation of NADH. To determine the IC_50_ of the small molecule inhibitors, varying concentration (2.5 nM to 10 µM) of compounds were incubated with 100 nM *Hp*IMPDH in assay buffer containing 50 mM Tris–HCl (pH 8.6), 100 mM KCl and 1 mM dithiothreitol (DTT). The assay mixture was incubated for 10 min at 25 °C and reaction was initiated by addition of NAD^+^ and IMP with a final concentration of 300 µM and 250 µM respectively. The production of NADH was monitored by measuring fluorescence at 440 nm when excited at 340 nm using PerkinElmer EnVision Multilabel Reader (Perkin Elmer, Inc., MA, USA) at 37 °C. In these assays C91 was used as a positive control.

IC_50_ values for all compounds were calculated with the help of software GraphPad Prism. The equation applied was:

2 $$Y\,={\rm{B}}{\rm{o}}{\rm{t}}{\rm{t}}{\rm{o}}{\rm{m}}+\frac{({\rm{T}}{\rm{o}}{\rm{p}}-{\rm{B}}{\rm{o}}{\rm{t}}{\rm{t}}{\rm{o}}{\rm{m}})}{1+{10}^{(({\rm{L}}{\rm{o}}{\rm{g}}{\rm{I}}{\rm{C}}50-{\rm{X}})\ast {\rm{H}}{\rm{i}}{\rm{l}}{\rm{l}}{\rm{S}}{\rm{l}}{\rm{o}}{\rm{p}}{\rm{e}})}}$$

### Conservation analysis of *Hp*IMPDH with other Prokaryotic and Eukaryotic IMPDHs

The amino acid sequences of some eukaryotic and prokaryotic IMPDH enzymes whose crystal structures^11,24,29,34–36^ are known were taken from the UniProt database^37,38^. These were namely, euk-IMPDH: *Homo sapiens* I (P20839), *Homo sapiens* II (P12268), *Ashbya gossypii* (Q756Z6), *Cricetulus griseus* (G3I4W7); pro-IMPDH: *Pseudomonas aeruginosa* (Q9HXM5), *Streptococcus pyogenes* (P0C0H6), *Escherichia coli* (P0ADG7), *Borrelia burgdorferi* (P49058) and *Cryptosporidium parvum* (Q8T6T2). Multiple sequence alignment of *Hp*IMPDH with eukaryotic and prokaryotic sequences were checked separately using a software Clustal Omega^39^. For better alignment, few initial amino acid residues were removed from the sequences. The results of Clustal Omega were analysed using Jalview software for the conservative and non-conservative residues^40^.

### Sequences retrieval and template selection for molecular model generation

The amino acid sequence of *Hp*IMPDH (ERM21512.1 IMP dehydrogenase) from *H. pylori* was retrieved from NCBI in FASTA format. This FASTA sequence was directly taken into Swiss model workspace for *Hp*IMPDH structure prediction. SWISS-MODE workspace was used to build three-dimensional protein structure of *Hp*IMPDH, using experimentally determined crystal structures of related family members as templates.

The target sequence was searched with BLAST and HHBlits^41^ for evolutionarily related structures matching the target sequence. Model generation of *Hp*IMPDH was carried out based on the target-sequence alignment using ProMod3. Visualisation and analysis of the model were done using the PyMOL and Maestro programmes^42^.

### Molecular Model evaluation

In this work, we have utilised PROCHEK^43^, ProSA^44^, RMSD and Ramachandran plot analysis to validate the final model. The PROCHECK online software was used to check the stereochemical quality of the protein by analysing residue by residue bond length, bond angles, torsional angles, chirality. ProSA online server was run to check the sequence to structure and validation of predicted 3-D structure. Ramachandran plot analysis was performed to visualise energetically allowed regions for backbone ϕ/ψ torsion angles.

### Molecular Docking

*In silico* docking studies were conducted using the Glide module (XP) of Schrödinger Maestro v11.2 software^42^. Docking consists of four steps: Protein Preparation^45^, Ligand Preparation^45^, Receptor Grid Generation, and Ligand Docking. For each docked ligand the best-docked pose with lowest Glide score value was recorded and compared.

## Supplementary information

Supporting information

## References

[1] Deen NS, Huang SJ, Gong L, Kwok T, Devenish RJ (2013). The impact of autophagic processes on the intracellular fate of Helicobacter pylori: more tricks from an enigmatic pathogen?. Autophagy.

[2] Thirumurthi S, Graham DY (2012). Helicobacter pylori infection in India from a western perspective. The Indian journal of medical research.

[3] Wroblewski LE, Peek RM, Wilson KT (2010). Helicobacter pylori and gastric cancer: factors that modulate disease risk. Clinical microbiology reviews.

[4] Rimbara E, Fischbach LA, Graham DY (2011). Optimal therapy for Helicobacter pylori infections. Nature reviews. Gastroenterology & hepatology.

[5] Megraud F (2004). H pylori antibiotic resistance: prevalence, importance, and advances in testing. Gut.

[6] Gollapalli DR (2010). Structural determinants of inhibitor selectivity in prokaryotic IMP dehydrogenases. Chemistry & biology.

[7] Cox JA (2016). Novel inhibitors of Mycobacterium tuberculosis GuaB2 identified by a target based high-throughput phenotypic screen. Sci Rep.

[8] Abrahamsen MS (2004). Complete genome sequence of the apicomplexan, Cryptosporidium parvum. Science.

[9] Jackson RC, Weber G, Morris HP (1975). IMP dehydrogenase, an enzyme linked with proliferation and malignancy. Nature.

[10] Zhang R (1999). Differential signatures of bacterial and mammalian IMP dehydrogenase enzymes. Current medicinal chemistry.

[11] Macpherson IS (2010). The structural basis of Cryptosporidium -specific IMP dehydrogenase inhibitor selectivity. Journal of the American Chemical Society.

[12] Kirubakaran S (2012). Structure-activity relationship study of selective benzimidazole-based inhibitors of Cryptosporidium parvum IMPDH. Bioorganic & medicinal chemistry letters.

[13] Hedstrom L, Liechti G, Goldberg JB, Gollapalli DR (2011). The Antibiotic Potential of Prokaryotic IMP Dehydrogenase Inhibitors. Current Medicinal Chemistry.

[14] Beevers RE (2006). Novel indole inhibitors of IMPDH from fragments: Synthesis and initial structure–activity relationships. Bioorganic & Medicinal Chemistry Letters.

[15] Hedstrom L (2009). IMP dehydrogenase: structure, mechanism, and inhibition. Chemical reviews.

[16] Kelly SM, Jess TJ, Price NC (2005). How to study proteins by circular dichroism. Biochimica et biophysica acta.

[17] Greenfield NJ (2006). Using circular dichroism spectra to estimate protein secondary structure. Nat Protoc.

[18] Sreerama N, Woody RW (2000). Estimation of protein secondary structure from circular dichroism spectra: comparison of CONTIN, SELCON, and CDSSTR methods with an expanded reference set. Anal Biochem.

[19] Whitmore L, Wallace BA (2008). Protein secondary structure analyses from circular dichroism spectroscopy: methods and reference databases. Biopolymers.

[20] Alexandre T, Raynal B, Munier-Lehmann H (2015). Two classes of bacterial IMPDHs according to their quaternary structures and catalytic properties. PloS one.

[21] Carr SF, Papp E, Wu JC, Natsumeda Y (1993). Characterization of human type I and type II IMP dehydrogenases. The Journal of biological chemistry.

[22] Hedstrom L (1999). IMP dehydrogenase: mechanism of action and inhibition. Current medicinal chemistry.

[23] Hedstrom L, Wang CC (1990). Mycophenolic acid and thiazole adenine dinucleotide inhibition of Tritrichomonas foetus inosine 5′-monophosphate dehydrogenase: implications on enzyme mechanism. Biochemistry.

[24] Sintchak MD (1996). Structure and mechanism of inosine monophosphate dehydrogenase in complex with the immunosuppressant mycophenolic acid. Cell.

[25] Digits JA, Hedstrom L (1999). Kinetic mechanism of Tritrichomonas foetus inosine 5′-monophosphate dehydrogenase. Biochemistry.

[26] Kim, Y., Makowska-Grzyska, M., Gu, M., Anderson, W.F., Joachimiak, A. X-ray diffraction data for the crystal Structure of the Inosine 5′-monophosphate Dehydrogenase with an Internal Deletion of the CBS Domain from Bacillus anthracis str. Ames complexed with inhibitor C91 (4MY9). *RSC PDB*, 10.18430/M34MY9 (2013).

[27] Maltseva, N. *et al*. Crystal Structure of Inosine 5′-monophosphate Dehydrogenase from Clostridium perfringens Complexed with IMP and C91. *RSC PDB*, 10.2210/pdb4q32/pdb (2014).

[28] Kim, Y. *et al*. Csgid, Center for Structural Genomics of Infectious Diseases. Crystal Structure of the Inosine 5′-monophosphate Dehydrogenase, with an Internal Deletion of CBS Domain from Campylobacter jejuni complexed with inhibitor compound C91, 10.2210/pdb4mz8/pdb (2013).

[29] Colby TD, Vanderveen K, Strickler MD, Markham GD, Goldstein BM (1999). Crystal structure of human type II inosine monophosphate dehydrogenase: implications for ligand binding and drug design. Proceedings of the National Academy of Sciences of the United States of America.

[30] Rostirolla DC (2014). D. S. Biochemical characterization of Mycobacterium tuberculosis IMP dehydrogenase: kinetic mechanism, metal activation and evidence of a cooperative system. RSC Advances.

[31] Biasini M (2014). SWISS-MODEL: modelling protein tertiary and quaternary structure using evolutionary information. Nucleic Acids Research.

[32] Gan L, Petsko GA, Hedstrom L (2002). Crystal structure of a ternary complex of Tritrichomonas foetus inosine 5′-monophosphate dehydrogenase: NAD+orients the active site loop for catalysis. Biochemistry.

[33] Li Q (2015). Identification of Novel Laminin- and Fibronectin-binding Proteins by Far-Western Blot: Capturing the Adhesins of Streptococcus suis Type 2. Frontiers in cellular and infection microbiology.

[34] Zhang R (1999). Characteristics and crystal structure of bacterial inosine-5′-monophosphate dehydrogenase. Biochemistry.

[35] Rao VA, Shepherd SM, Owen R, Hunter WN (2013). Structure of Pseudomonas aeruginosa inosine 5′-monophosphate dehydrogenase. *Acta crystallographica*. Section F, Structural biology and crystallization communications.

[36] McMillan FM (2000). Crystal structure at 2.4 A resolution of Borrelia burgdorferi inosine 5′-monophosphate dehydrogenase: evidence of a substrate-induced hinged-lid motion by loop 6. Biochemistry.

[37] UniProt C (2015). UniProt: a hub for protein information. Nucleic acids research.

[38] Magrane M, UniProt C (2011). UniProt Knowledgebase: a hub of integrated protein data. Database: the journal of biological databases and curation.

[39] Sievers F (2011). Fast, scalable generation of high-quality protein multiple sequence alignments using Clustal Omega. Molecular systems biology.

[40] Waterhouse AM, Procter JB, Martin DM, Clamp M, Barton GJ (2009). Jalview Version 2–a multiple sequence alignment editor and analysis workbench. Bioinformatics.

[41] Remmert M, Biegert A, Hauser A, Soding J (2011). HHblits: lightning-fast iterative protein sequence searching by HMM-HMM alignment. Nat Methods.

[42] Friesner RA (2006). Extra Precision Glide:  Docking and Scoring Incorporating a Model of Hydrophobic Enclosure for Protein−Ligand Complexes. Journal of Medicinal Chemistry.

[43] Laskowski RA, MacArthur MW, Moss DS, Thornton JM (1993). PROCHECK: a program to check the stereochemical quality of protein structures. Journal of Applied Crystallography.

[44] Wiederstein M, Sippl MJ (2007). ProSA-web: interactive web service for the recognition of errors in three-dimensional structures of proteins. Nucleic Acids Research.

[45] Madhavi Sastry G, Adzhigirey M, Day T, Annabhimoju R, Sherman W (2013). Protein and ligand preparation: parameters, protocols, and influence on virtual screening enrichments. Journal of Computer-Aided Molecular Design.

