# Supplementary material for: Identification of selective inhibitors of Helicobacter pylori IMPDH as a targeted therapy for the infection
Source: Sci Rep. 2019 Jan 17;9:190. doi: 10.1038/s41598-018-37490-x (PMC6336804; doi:10.1038/s41598-018-37490-x)
Supplement: Supplementary file 1 — Supporting information [file 41598_2018_37490_MOESM1_ESM.docx]

**Supporting Information**

**Identification of selective inhibitors of *Helicobacter pylori* IMPDH as a targeted therapy for the infection**

Kapil Juvale^1,4,#^, Gayathri Purushothaman^2,#^, Vijay Singh^2^, Althaf Shaik^1^, Srimadhavi Ravi^1^, Vijay Thiruvenkatam^2,3^, Sivapriya Kirubakaran^1,2^*

^1^ Chemistry, Indian Institute of technology Gandhinagar, Palaj Village, Gandhinagar, India-382355

^2^ Bio-engineering, Indian Institute of technology Gandhinagar, Palaj Village, Gandhinagar, India-382355

^3^ Physics, Indian Institute of technology Gandhinagar, Palaj Village, Gandhinagar, India-382355

^4^ Shobhaben Pratapbhai Patel School of Pharmacy & Technology Management, SVKM’s NMIMS, V.L. Mehta Road, Vile Parle (W), Mumbai, India-400056

^#^ Equal Contribution

* correspondence: Dr. Sivapriya Kirubakaran

Chemistry and Bio-engineering, Indian Institute of technology Gandhinagar, Palaj Village, Gandhinagar, India-382355;

[priyak@iitgn.ac.in](mailto:priyak@iitgn.ac.in).

Table of Contents

|  | Chemicals and materials | S3 |
| --- | --- | --- |
|  | Cloning of *Hp*IMPDH gene | S3 |
|  | Expression and Purification of *Hp*IMPDH | S3 |
|  | Figure S1 | S5 |
|  | Figure S2 | S5 |
|  | Table S1 | S6 |
|  | Table S2 | S6 |
|  | Figure S3 | S9 |
|  | Figure S4 | S10 |
|  | Figure S5 | S11 |
|  | Figure S6 | S12 |
|  | Figure S7 | S13 |
|  | Table S3 | S14 |
|  | Table S4 | S15 |
|  | Figure S8. | S15 |
|  | Table S5 | S16 |
|  | Table S6 | S16 |
|  | Figure S9 | S17 |
|  | Homology model of *Hp*IMPDH | S18 |
|  | Figure S10 | S18 |
|  | Figure S11 | S19 |
|  | Molecular Model validation | S20 |
|  | Figure S12 | S20 |
|  | Figure S13 | S21 |
|  | *In silico* docking studies | S21 |
|  | Figure S14 | S22 |
|  | Figure S15 (Full SDS-page Gel) | S23 |
|  | Figure S16 (Western Blots) | S24 |
|  | Refereces | S25 |

Experimental Procedures

**Chemicals and materials**

The wild type *Hp*IMPDH protein (WP_022576538.1) consisting of397 amino acids residues is encoded by 1491bp long nucleotide sequence. This sequence was codon optimized for the *E.coli* expression system. The gene sequence cloned into pUC57 between the restriction enzyme site NotI and EcoRI was procured from Genscript,USA and the gene specific primers procured from sigma Aldrich. The bacterial strains DH5α and Rosetta™(DE3) pLysS were procured from Invitrogen. Chemicals like IMP, NAD, DMSO, IPTG, LB, Kanamycin, Triton-X, Glycerol were purchased from Sigma Aldrich. Restriction enzymes, Ligase, DNA polymerase were bought from NEB.

**Cloning of *Hp*IMPDH gene:**

The *Helicobacter pylori (H. p*ylori*)* IMPDH amino acid sequence was obtained from NCBI (WP_022576538.1). The protein sequence was reverse translated using the online tool Expasy and codon optimized for *E.coli* expression system. The synthetic *Hp*IMPDH gene in pUC57, was amplified using the gene specific primers (**FP**:5’-GGAGAATTCATGAGAATTTTACAAAGG-3’ and **RP**:5’-GTAGCGGCCGCTTACCCATAATAATTAG-3’). The resultant amplified product was cloned into expression vector pET-28a(+) between the restriction enzyme site EcoRI and NotI. Following this, the recombinant vector was used to transform the DH5α cells and the positive clones were selected on LB medium containing 50µg/mL Kanamycin and the clones were confirmed by restriction digestion. The cloned gene was further analyzed by DNA sequencing to confirm that there are no mutations present.

**Expression and Purification of *Hp*IMPDH**

The recombinant plasmid pET-28a(+) containing *Hp*IMPDH gene was used to transform Rosetta™(DE3) pLysS expression cell strain and the cells having recombinant plasmids were selected on LB medium containing Kanamycin. To check the expression of recombinant *Hp*IMPDH, single transformed colony was used to inoculate a primary culture of 10 mL LB media containing Kanamycin and the culture was grown at 37°C, 250rpm for 12hrs.The secondary inoculation was done in 100 ml media with 1% inoculum of primary culture. This culture was grown at 37°C, 250rpm until it reached the OD_600_ of 0.6 to 0.8. The cells were then induced with 0.5mM IPTG and grown at 25°C. After 12hrs of growth 220 rpm, the cells were harvested by centrifugation at 7800rpm for 15min at 4°C and the pellet was re-suspended in cell lysis buffer (40mM Tris-Cl (pH7.8), 300mM NaCl,1mM β-Me,1% Triton-X 100 and 2Mm PMSF), followed by sonication at 40% amplitude (10 sec ON and 15 sec OFF cycle for 15 min). The cell lysate was then centrifuged at 7800 rpm for 45 min at 4 °C and the protein expression was checked in both supernatant and pellet.

The recombinant *Hp*IMPDH protein was purified using Ni-NTA resin in a gravity flow column (Bio-Rad). The column was equilibrated with 10 column volumes (CV) of Buffer I (40mM Tris-Cl (pH7.8) and 300mM NaCl) at a flow rate of 1mL/min. The supernant containing the recombinant *Hp*IMPDH was loaded onto the equilibrated column. The unbound fraction was allowed to flow through at a flow rate of 1mL/min following which the column was washed with 30 CV of Buffer II (40mMTris-Cl (pH7.8), 300mM NaCl and 20mM Imidazole (pH 7.8). The recombinant *Hp*IMPDH was eluted using 5CV of Buffer III (40mM Tris-Cl (pH7.8), 300mM NaCl, 10%Glycerol and 300mM Imidazole (pH7.8) at a flow rate of 0.3mL/min. All the eluted fractions were analyzed by12% SDS-PAGE .The relatively pure fractions were pooled and desalted using 10kDa Amicon (Merck) followed by further purification by size exclusion chromatography (FPLC-Sephadex-75). The purified protein was concentrated by 10kDa Amicon centrifugal filters and the concentration of the purified protein was determined by performing Bradford assay (Bio-Rad dye). The presence of recombinant His tagged *Hp*IMPDH protein was confirmed by western blot using primary His-Tag antibody (Rabbit monoclonal, Cell Signalling Technology) and HRP linked secondary antibody (Anti-rabbit IgG, Cell signaling Technology) [^1^](#_ENREF_1).

Results and Discussion

**
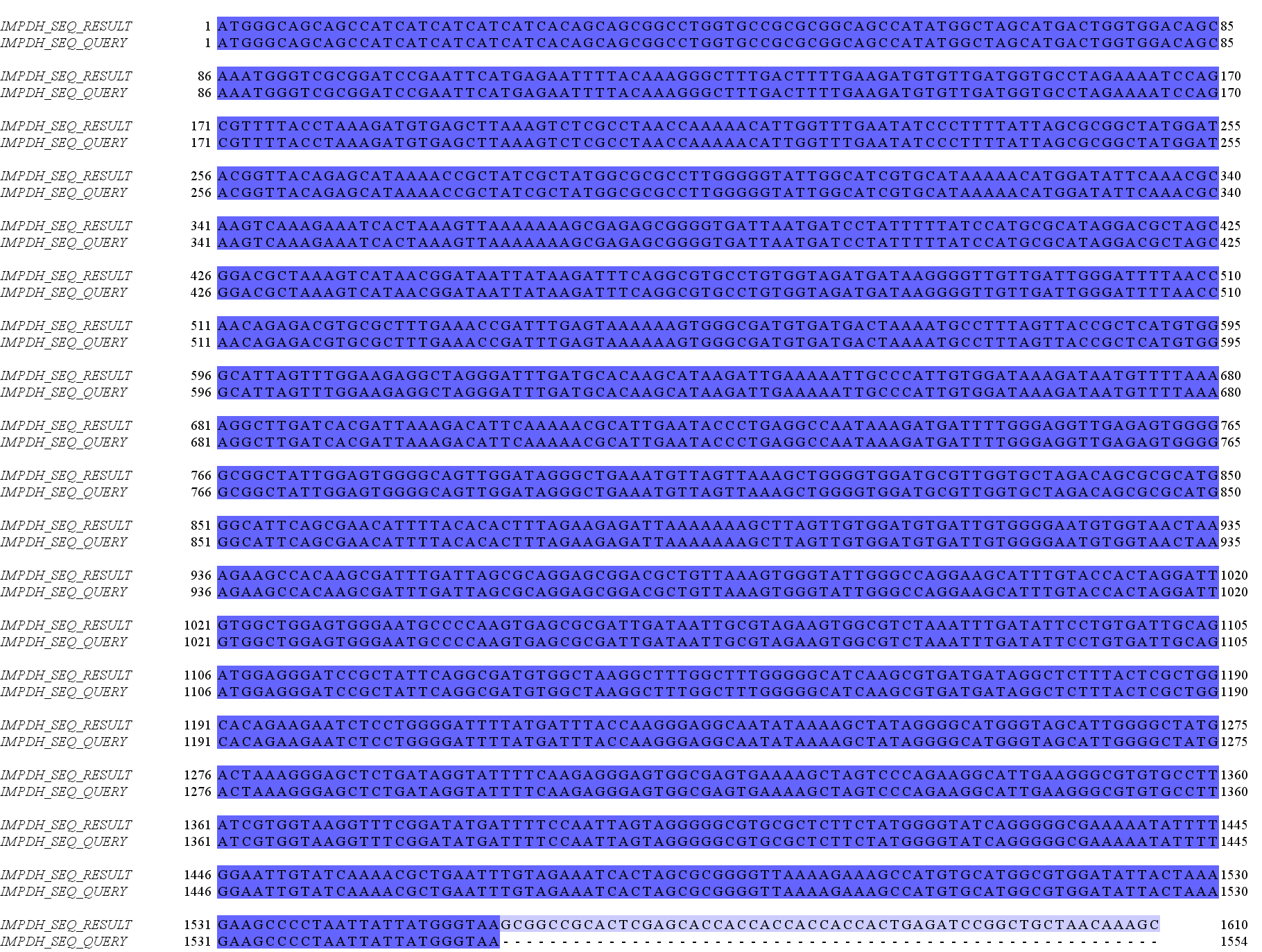
**

**Figgure S1.** DNA sequencing result after cloning of IMPDH sequence into pET28a

IMPDH_SEQ_RESULT: Nucleotide sequence obtained from DNA sequencing

IMPDH_SEQ_QUERY: Sequence that cloned into pET28a


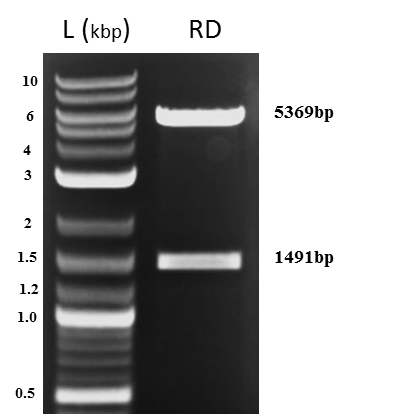


**Figure S2.** Confirmation of the cloning by restriction digestion with NotI and the EcoRI; the lane labelled L has the 10 Kbp DNA ladder and RD has double digested product of pET28a which contains the IMPDH gene.

**Table S1.** Resultant LC-MS/MS parameters for *Hp*IMPDH from MOSCOT software

| Accession no | WP_001223726.1 |
| --- | --- |
| Protein name | IMP dehydrogenase |
| Organism | *Helicobacter pylori* |
| Mascot score | 88 |
| Coverage | 65 |
| Sequence similarity between HpIMPDH of WP_001223726.1* and WP_022576538.1** | 99% |

**** WP_022576538.1 - Accession no of *Hp*IMPDH amino acid sequence obtained from NCBI;

*WP_001223726.1 – Resultant Accession no. obtained from Moscot.

**Table S2.** Mass analysis of the purified recombinant *Hp*IMPDH

| m/z | S/N | Quality Fac. | Res. | Intens. | Area |
| --- | --- | --- | --- | --- | --- |
| 529.402 | 26 | 3527 | 8672 | 3477 | 337 |
| 532.549 | 20 | 6320 | 8816 | 2727 | 262 |
| 534.37 | 43 | 21292 | 9365 | 5819 | 543 |
| 548.556 | 11 | 975 | 9132 | 1523 | 149 |
| 550.209 | 8 | 1859 | 9125 | 1105 | 104 |
| 568.22 | 52 | 55025 | 9014 | 6963 | 699 |
| 588.66 | 7 | 718 | 10138 | 880 | 85.8 |
| 606.32 | 16 | 4486 | 10889 | 2124 | 197 |
| 614.685 | 7 | 1551 | 11009 | 959 | 86.3 |
| 634.352 | 83 | 16058 | 11139 | 11118 | 1067 |
| 642.275 | 6 | 750 | 7685 | 842 | 124 |
| 642.712 | 24 | 4972 | 11398 | 3294 | 311 |
| 644.113 | 8 | 1475 | 8291 | 1054 | 146 |
| 650.127 | 36 | 42992 | 11942 | 4839 | 445 |
| 656.143 | 6 | 837 | 9833 | 809 | 94.4 |
| 656.334 | 7 | 1227 | 6870 | 908 | 159 |
| 666.101 | 34 | 18016 | 11969 | 4673 | 443 |
| 672.115 | 7 | 2034 | 10813 | 969 | 110 |
| 682.072 | 10 | 3687 | 13669 | 1385 | 120 |
| 726.4 | 9 | 1798 | 7368 | 1246 | 223 |
| 776.324 | 6 | 1099 | 11103 | 882 | 116 |
| 804.361 | 110 | 31845 | 12527 | 15057 | 1751 |
| 806.358 | 9 | 2747 | 10934 | 1285 | 189 |
| 826.34 | 8 | 2272 | 13368 | 1089 | 130 |
| 842.312 | 7 | 1881 | 13161 | 971 | 120 |
| 842.589 | 7 | 2993 | 12126 | 1009 | 133 |
| 855.138 | 7 | 3275 | 13701 | 1033 | 124 |
| 861.152 | 12 | 13521 | 13772 | 1608 | 195 |
| 864.291 | 7 | 1083 | 12500 | 968 | 139 |
| 877.13 | 18 | 12252 | 14045 | 2517 | 295 |
| 893.101 | 9 | 1799 | 15386 | 1294 | 147 |
| 969.614 | 31 | 28921 | 14531 | 4408 | 590 |
| 990.641 | 38 | 21193 | 15183 | 5398 | 730 |
| 1047.662 | 16 | 20211 | 15091 | 2328 | 347 |
| 1061.773 | 11 | 5967 | 1574 | 1585 | 2090 |
| 1069.757 | 130 | 147801 | 15554 | 18823 | 2788 |
| 1091.744 | 14 | 13009 | 14015 | 1968 | 327 |
| 1107.715 | 7 | 507 | 9796 | 979 | 240 |
| 1155.734 | 42 | 62208 | 14724 | 6200 | 1110 |
| 1217.716 | 14 | 11541 | 14655 | 2120 | 416 |
| 1272.782 | 279 | 184965 | 14117 | 42072 | 9448 |
| 1286.804 | 8 | 2879 | 12225 | 1253 | 318 |
| 1294.765 | 7 | 2323 | 10446 | 1122 | 358 |
| 1342.83 | 7 | 3596 | 13616 | 1120 | 296 |
| 1353.65 | 18 | 9164 | 3585 | 2818 | 2672 |
| 1383.649 | 28 | 13240 | 3785 | 4311 | 4005 |
| 1390.831 | 74 | 129206 | 13536 | 11443 | 3149 |
| 1403.849 | 7 | 221 | 15807 | 1112 | 262 |
| 1406.825 | 73 | 139824 | 12669 | 11335 | 3430 |
| 1420.856 | 91 | 104376 | 13036 | 14099 | 4166 |
| 1436.852 | 79 | 132881 | 12200 | 12214 | 3943 |
| 1452.847 | 9 | 11369 | 12468 | 1425 | 466 |
| 1463.955 | 50 | 51998 | 11392 | 7698 | 2633 |
| 1479.933 | 37 | 56298 | 12283 | 5679 | 1854 |
| 1553.83 | 55 | 201716 | 13074 | 8579 | 2957 |
| 1567.842 | 10 | 7024 | 9012 | 1605 | 838 |
| 1685.599 | 20 | 7606 | 3921 | 3087 | 4141 |
| 1709.937 | 38 | 119855 | 11334 | 5904 | 2842 |
| 1723.038 | 44 | 111812 | 12262 | 6797 | 3025 |
| 1739.028 | 50 | 202865 | 11950 | 7773 | 3693 |
| 1755.033 | 12 | 16501 | 11809 | 1845 | 893 |
| 1761.012 | 7 | 2185 | 9563 | 1026 | 622 |
| 1768.961 | 31 | 101514 | 12872 | 4775 | 2235 |
| 1792.031 | 174 | 342856 | 12692 | 26904 | 12758 |
| 1806.049 | 25 | 44101 | 12456 | 3842 | 1872 |
| 1810.98 | 57 | 116781 | 13061 | 8725 | 4177 |
| 1920.134 | 63 | 270172 | 12666 | 9277 | 4965 |
| 2079.354 | 14 | 39894 | 11811 | 1794 | 1126 |
| 2133.538 | 7 | 3548 | 3900 | 846 | 1616 |
| 2171.26 | 14 | 2509 | 7317 | 1664 | 1854 |
| 2187.25 | 37 | 133994 | 11379 | 4520 | 3136 |
| 2209.236 | 8 | 3564 | 10236 | 976 | 776 |
| 2252.332 | 57 | 124893 | 11519 | 6577 | 4636 |
| 2274.31 | 29 | 33185 | 12260 | 3277 | 2262 |
| 2290.294 | 21 | 51507 | 12304 | 2382 | 1618 |
| 2807.486 | 16 | 8700 | 10831 | 1192 | 1267 |
| 2838.702 | 107 | 108073 | 10672 | 7823 | 8380 |
| 2860.672 | 16 | 8759 | 10327 | 1125 | 1293 |
| 2876.652 | 11 | 4991 | 10785 | 783 | 855 |
| 3066.646 | 12 | 5580 | 4085 | 720 | 2142 |
| 3103.689 | 27 | 4481 | 5965 | 1526 | 3506 |
| 3119.683 | 20 | 3871 | 5320 | 1160 | 2749 |
| 3141.758 | 23 | 2634 | 8264 | 1311 | 2214 |
| 3157.767 | 31 | 6664 | 8733 | 1765 | 2825 |
| 3173.783 | 29 | 12186 | 9124 | 1620 | 2458 |

**
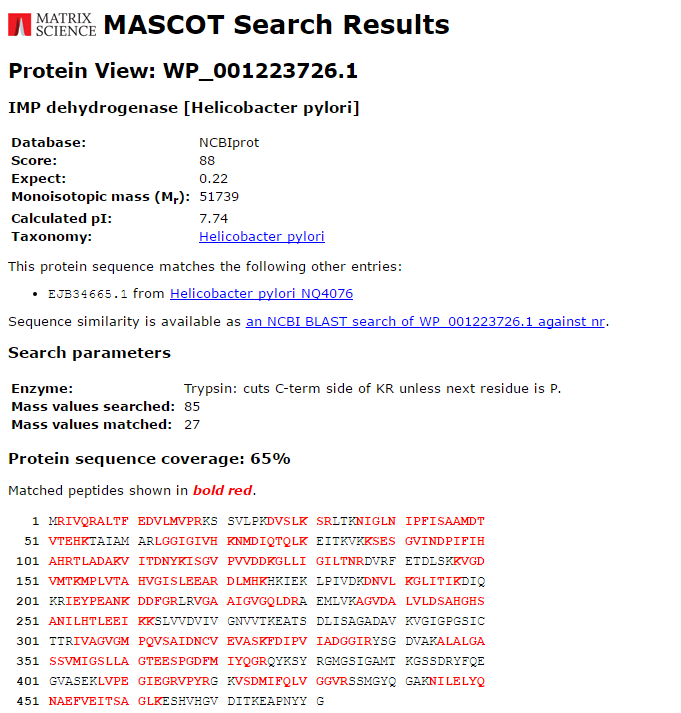
**

**Figure S3.** Peptide mass finger printing result from MASCOT software


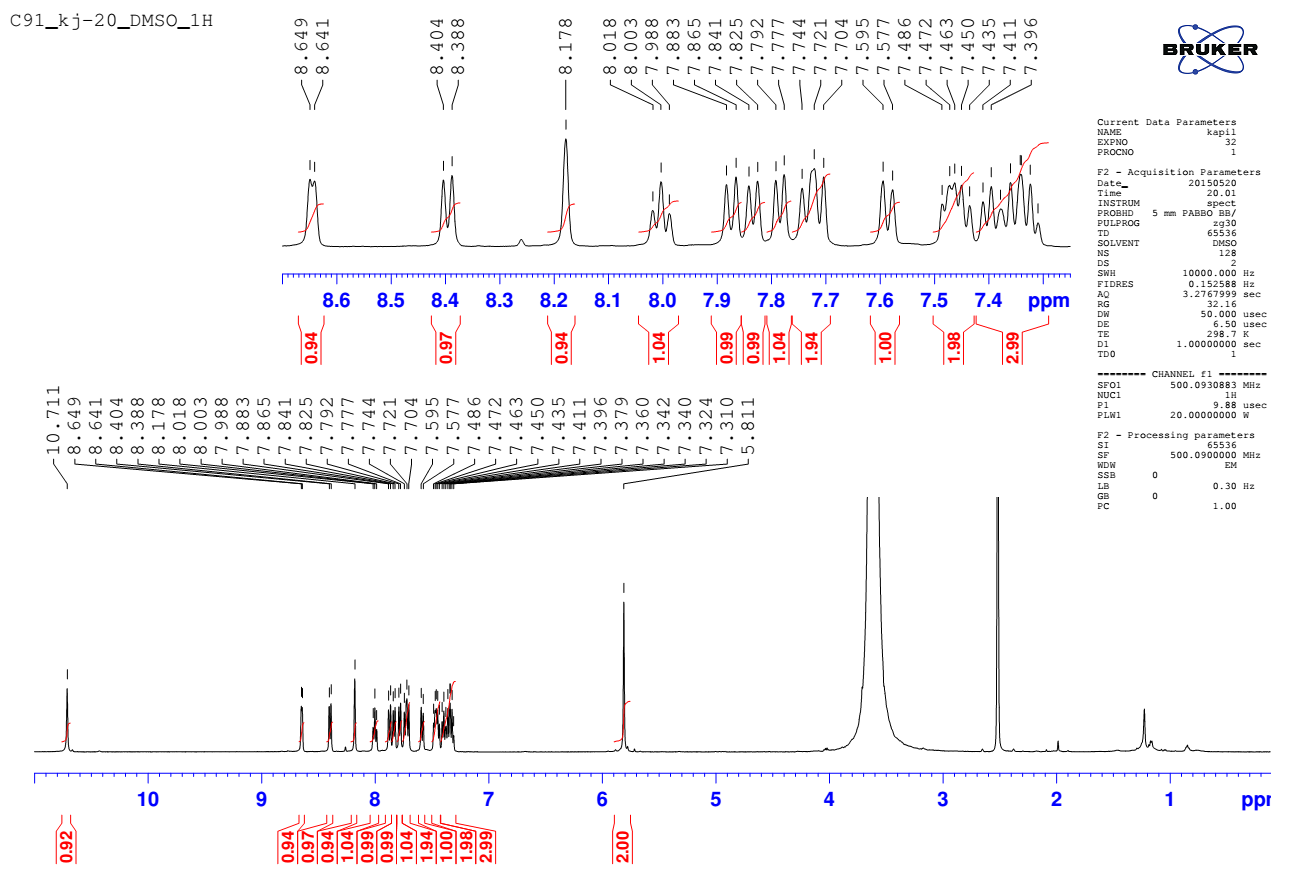


**Fig. S4:** ^1^H NMR of C91 **(1)**


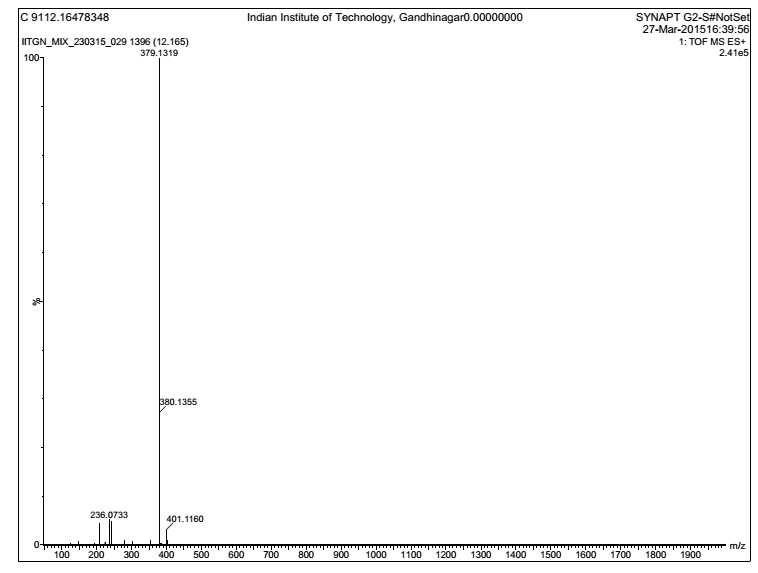


**Fig. S5:** Mass spectra of **C91 (1)**


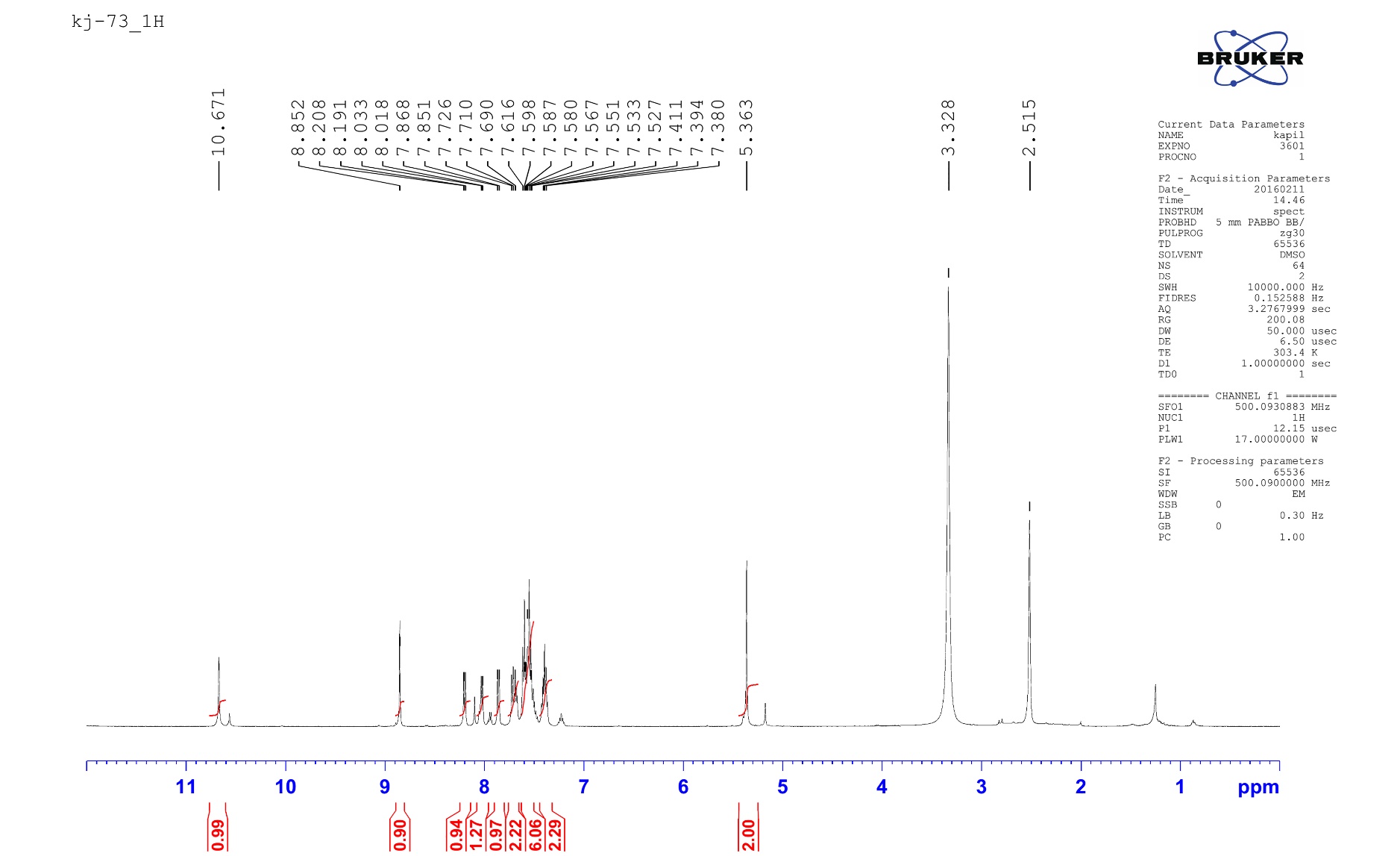


**Fig. S6:** ^1^H NMR of **2**


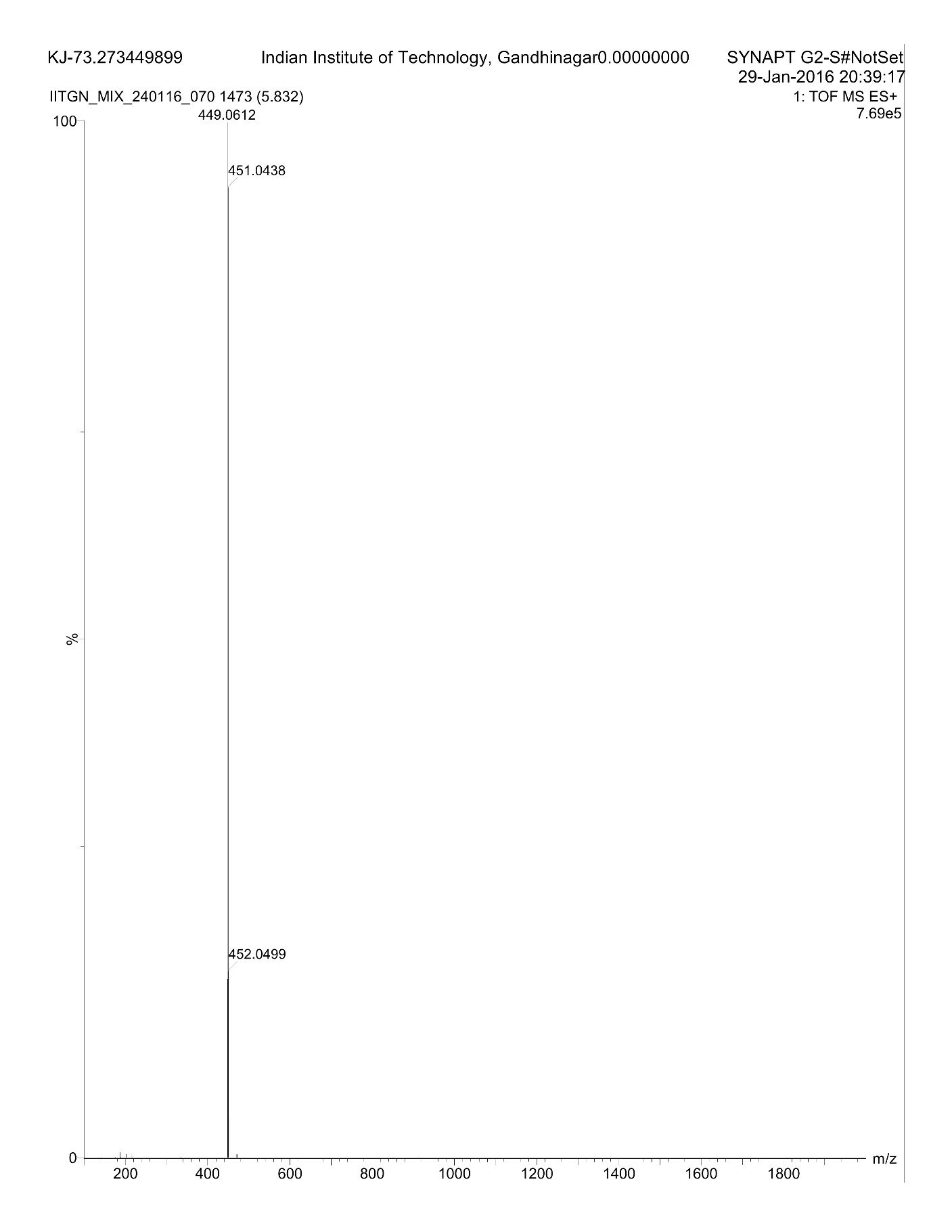


**Fig. S7:** Mass Spectra of **2**

**Table S3.** Inhibition profile of Indole based compounds

| **Compound** | **Compound** | **IC_50_ (M)** |
| --- | --- | --- |
| **2** |  | 1.88 x 10 ^-6^ |
| **3** |  | 5.628 x 10 ^-6^ |
| **4** |  | No inhibition |
| **5** |  | 9.707 x 10 ^-6^ |
| **6** |  | 3.019 x 10 ^-6^ |
| **7** |  | 14.44 x 10 ^-6^ |
| **8** |  | 30.88 x 10 ^-6^ |

**Table S4.** Comparison of effect of different concentrations of selected small molecules on the Km and Vmax values of *Hp*IMPDH for IMP and NAD^+^

| **Compound** | | | **1** | **2** |
| --- | --- | --- | --- | --- |
| 0µM | IMP | Vmax | 0.5117 | 0.658 |
|  |  | Km | 18.54 | 18.14 |
|  | NAD^+^ | Vmax | 1.156 | 0.928 |
|  |  | Km | 69.7 | 70.71 |
| 0.025µM | IMP | Vmax | 0.3607 | 0.5218 |
|  |  | Km | 12.95 | 17.95 |
|  | NAD^+^ | Vmax | 0.468 | 0.4511 |
|  |  | Km | 37.6 | 45.67 |
| 0.05µM | IMP | Vmax | 0.2919 | 0.4959 |
|  |  | Km | 10.29 | 18.21 |
|  | NAD^+^ | Vmax | 0.3956 | 0.3625 |
|  |  | Km | 33.11 | 44.93 |
| 0.1µM | IMP | Vmax | 0.2481 | 0.429 |
|  |  | Km | 09.49 | 17.94 |
|  | NAD^+^ | Vmax | 0.3263 | 0.3332 |
|  |  | Km | 26.32 | 45.18 |
| Conclusion | IMP | | Un-competitive inhibition | Non-competitive inhibition |
|  | NAD^+^ | | Un-competitive inhibition | Non-competitive inhibition |

**Figure S8.** Human IMPDH(II) enzyme inhibition of selected small molecules (10 μM). MPA (mycophenolic acid) was used as a standard. The data is normalized by considering untreated enzyme activity as 100%.

**Table S5.** Secondary structure elements of *Hp*IMPDH and IMPDHs from other organisms

| Protein | | Helix | Strand | Turns | Others | Total |
| --- | --- | --- | --- | --- | --- | --- |
| *Hp*IMPDH | | 37 | 17 | 7 | 39 | 100 |
| CD Spectrum derived secondary structure | *Hs*IMPDH-II[^2^](#_ENREF_2) | 30 | 24 | 18 | 28 | 100 |
|  | *Cg*IMPDH | 30 | 24 | 18 | 28 | 100 |
| Crystal structure derived secondary structure | *Hs*IMPDH-II | 34 | 16 | - | 50 | 100 |
|  | *Ag*IMPDH | 35 | 15 | - | 55 | 100 |
|  | *Pa*IMPDH | 22 | 9 | - | 69 | 100 |
|  | *Sp*IMPDH | 33 | 20 | - | 47 | 100 |
|  | *Bb*IMPDH | 28 | 14 | - | 58 | 100 |
|  | *Cp*IMPDH | 25 | 16 | - | 59 | 100 |

[Ag- *Ashbya gossypii* [PDB:4Z0G],Cg-*Cricetulus griseus*, *Hs-Homo sapience* [PDB:1B3O], *Pa-Pseudomonas aeruginosa* [PDB:3ZFH],*Sp-Streptococcus pyrogenus* [PDB:1ZFJ], *Bb-Borrelia burgdorferi* [PDB:1EEP],*Cp-Cryptosporidium parvum* [PDB:3FFS].

**Table S6.** Kinetics parameters of *Hp*IMPDH and other reported IMPDHs

| **Protein** | **K_M_ (µM)** | | **Kcat(s-1)** |
| --- | --- | --- | --- |
|  | **IMP** | **NAD** |  |
| *Hp*IMPDH | 18.36 ± 1.53 | 76.37 ± 1.09 | 5.57 ± 0.88 |
| *Hp*IMPDH[^3^](#_ENREF_3)‡ | 18 ± 3 | 73 ± 6 | 3.0 ± 0.3 |
| **Eukaryotic IMPDH** | | | |
| *Hs*IMPDH-I[^4^](#_ENREF_4) | 9.3 ± 1.0 | 32 ± 3.4 | 1.3 ± 0.00 |
| *Hs*IMPDH-II[^4^](#_ENREF_4) | 18 ± 2.2 | 46 ± 2.8 | 1.5 ± 0.10 |
| *Ag*IMPDH[^5^](#_ENREF_5) | 90.2 ± 5.6 | 279.8 ± 24.7 | 2.46 ± 0.04 |
| *TfI*MPDH[^6^](#_ENREF_6) | 1.7± 0.4 | 150 ± 30 | 1.9 ± 0.2 |
| **Prokaryotic IMPDH** | | | |
| *PaI*MPDH[^7^](#_ENREF_7) | 139 ± 14 | 1760 ± 109 | NA |
| *Cp*IMPDH[^8^](#_ENREF_8) | 29 ± 3 | 150 ± 20 | 3.3 ± 0.2 |
| *SpI*MPDH[^9^](#_ENREF_9) | 62 ±19 | 1180 ± 400 | 24 ± 3 |
| *BbI*MPDH[^10^](#_ENREF_10) | 29 ± 8 | 1100 ± 160 | 2.6 ±0.3 |

‡ previously reported *Hp*IMPDH data


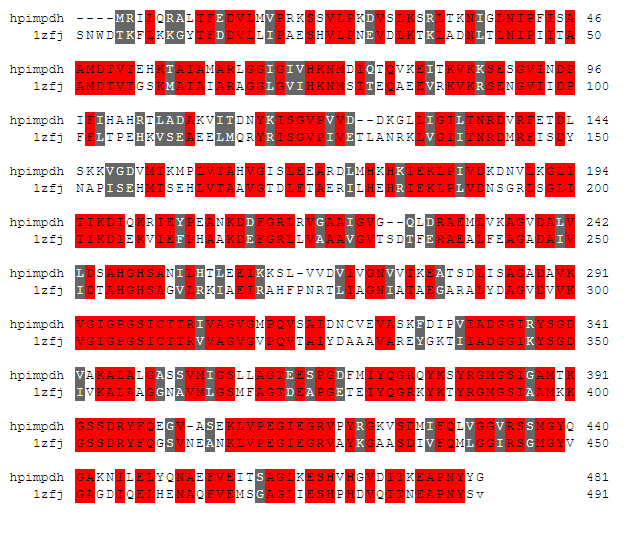


**Figure S9.** Pair wise alignment of IMPDH from H.pylori and the template PDB; 1ZFJ (highlighting the conserved residue in red colour)

**Homology model of *Hp*IMPDH**

The template structure 1ZFJ has the IMP ligand, which is transferred into the putative structure of HpIMPDH model using the SWISS-MODEL[^11^](#_ENREF_11). The residues comprising of active site of SpIMPDH are Ser 307, Ser 366, Gly 344, Gly 365, Tyr 389, Gly 309, Met 392 and Asp 342. The corresponding residues (Ser 298, Ser 357, Gly 335, Gly 356, Tyr 380, Gly 300, Met 383 and Asp 333) of HpIMPDH active site were identified by sequence alignment and visualised in PyMol ( Fig.S11.).

**
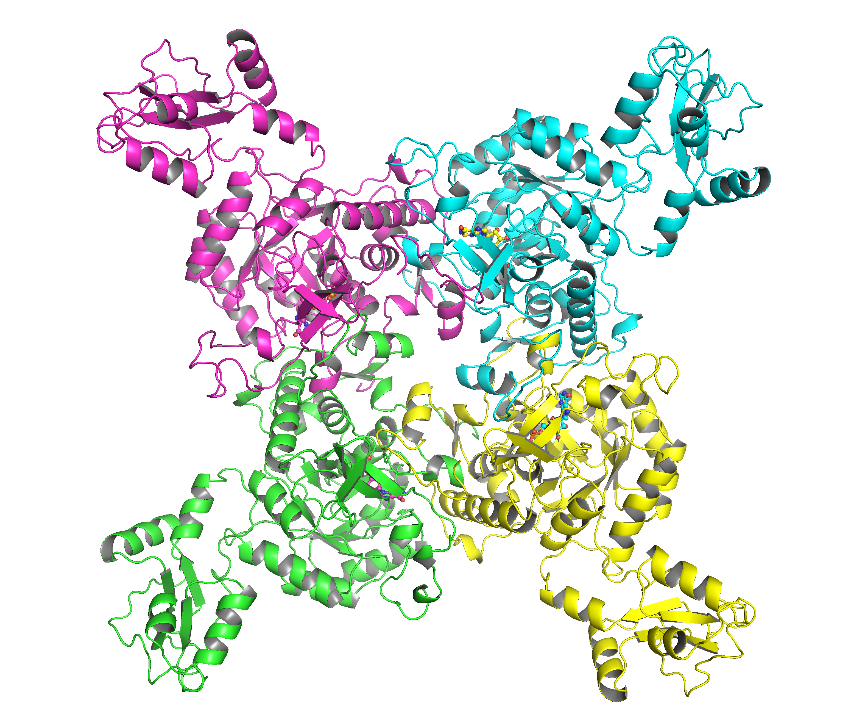
**

**Figure S10.** Modelled structure of IMPDH from *H.pylori*


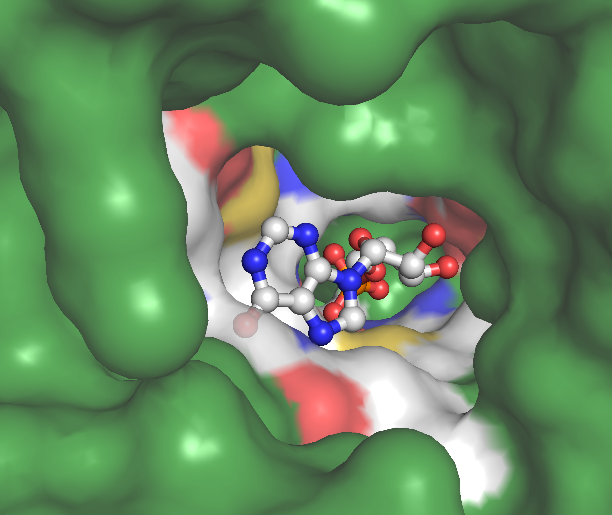

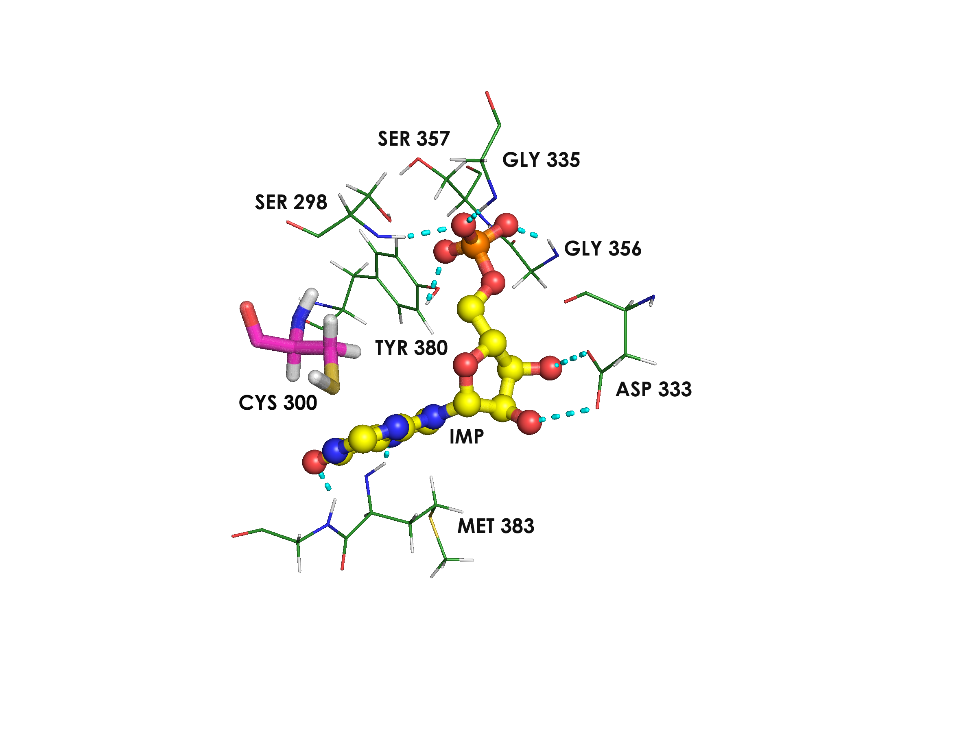


**Figure S11.** IMP binding pocket in predicted structure of IMPDH from *H.pylori*, highlighting the key residues in the pocket

**Molecular Model validation**

The root mean square deviation (RMSD) analysis was carried out by superposing 1ZFJ over generated model (i.e. the back bone atoms of alpha carbon) to check the accuracy and reliability of the predicted model using superpose. The Cα RMSD and the backbone RMSD deviations for the model and the template crystal structure are 0.88 Å and 0.88 Å respectively.

Stereochemical quality evolution and overall assessment of the structure geometry of the predicted structure was analysed using Procheck program. The predicted structure satisfied stereo chemical restraints and passed all criteria implemented in Procheck.

Generated IMPDH structure were evaluated from energetics and geometric criteria with ProSA online server. ProSA online server calculate the z-score, which specify the quality of the protein structure. The z-score of the predicated and template 1ZFJ are -9.04 and -9.35 (Fig.S11). In general, the z-score should be below the zero for no significant stressed or stained folds with high energies. Also majority of peaks of the predicted model are below the peaks of 1ZFJ structure in the overlap energy profile graph, which shows the quality of predicted *Hp*IMPDH.


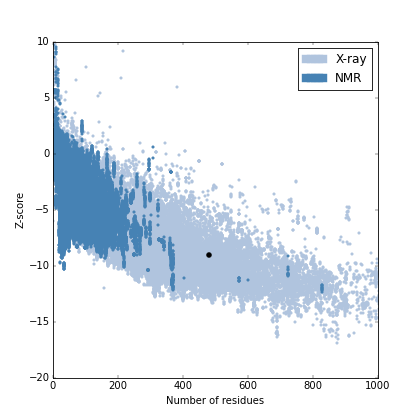

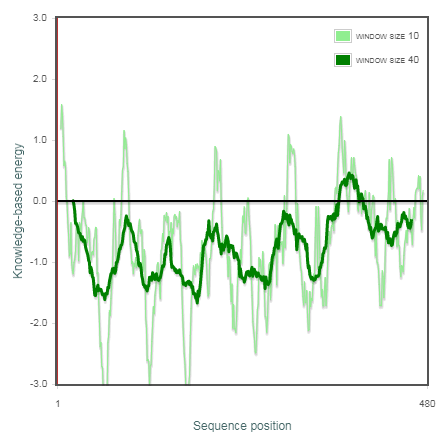


**Figure S12.** The Z-score vs protein residue position for predicted *H.pylori* IMPDH

Ramachandran plot analysis (Fig. S13) revealed 93.7 % residues of modelled structure in the favored region and 5.2 % residues in the allowed region. Apart from that, 1.1 % residue were in the disallowed region (Fig.S12.). The predicted structure was further subjected to energy minimization by GROMOS 96 force field, implemented in Swiss PDB viewer software. Energy minimization was performed (100 steps steepest descent followed by 200 steps conjugate gradient).The generated 3D structure was deposited at the Protein Model Database (PMDB) and assigned the PMDB ID:  PM0081355.

Based on the results obtained from PROCHECK, RMSD and ProSA programme, the predicated *H.pylori* IMPDH model is reasonable and reliable for performing in silico docking studies.


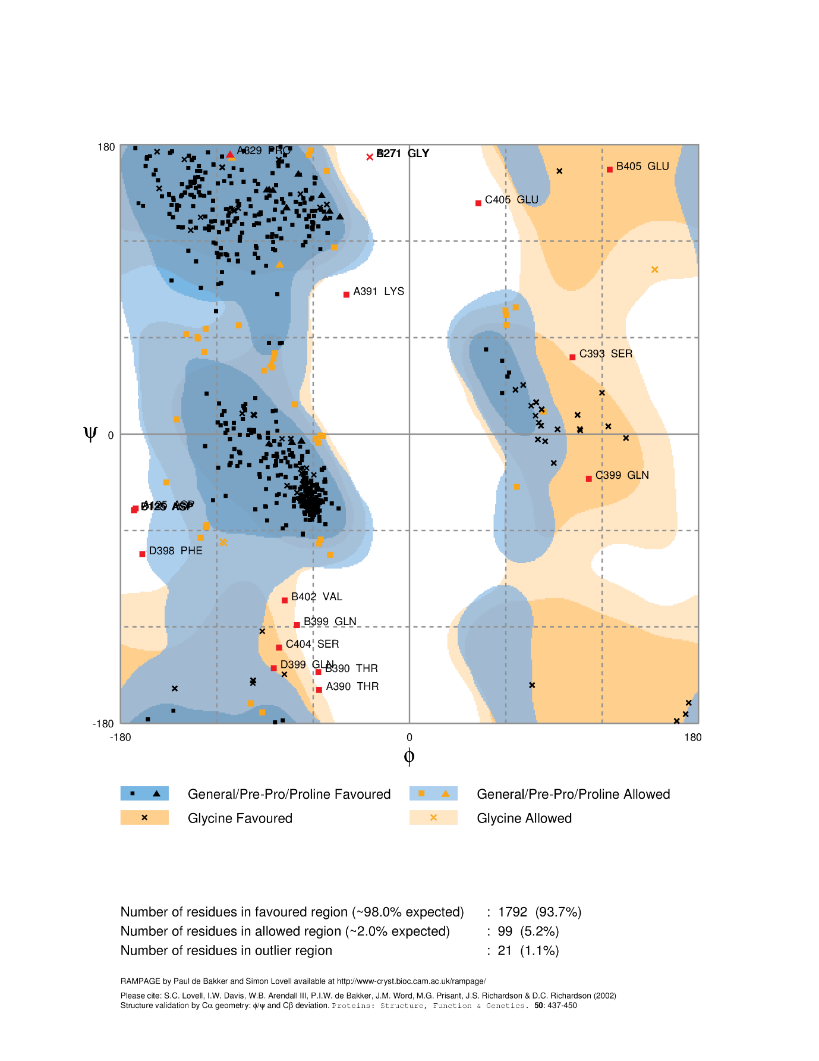


**Figure S13.** Ramachandran plot for predicted *HpIMPDH* structure

***In silico* docking studies**

*In silico* docking studies were conducted using the Glide module (XP) of Schrödinger Maestro v11.2 software[^12^](#_ENREF_12). Docking consists of four steps: Protein Preparation[^13^](#_ENREF_13), Ligand Preparation[^13^](#_ENREF_13), Receptor Grid Generation, and Ligand Docking. For each docked ligand the best docked pose with lowest Glide score value was recorded and compared.

1.
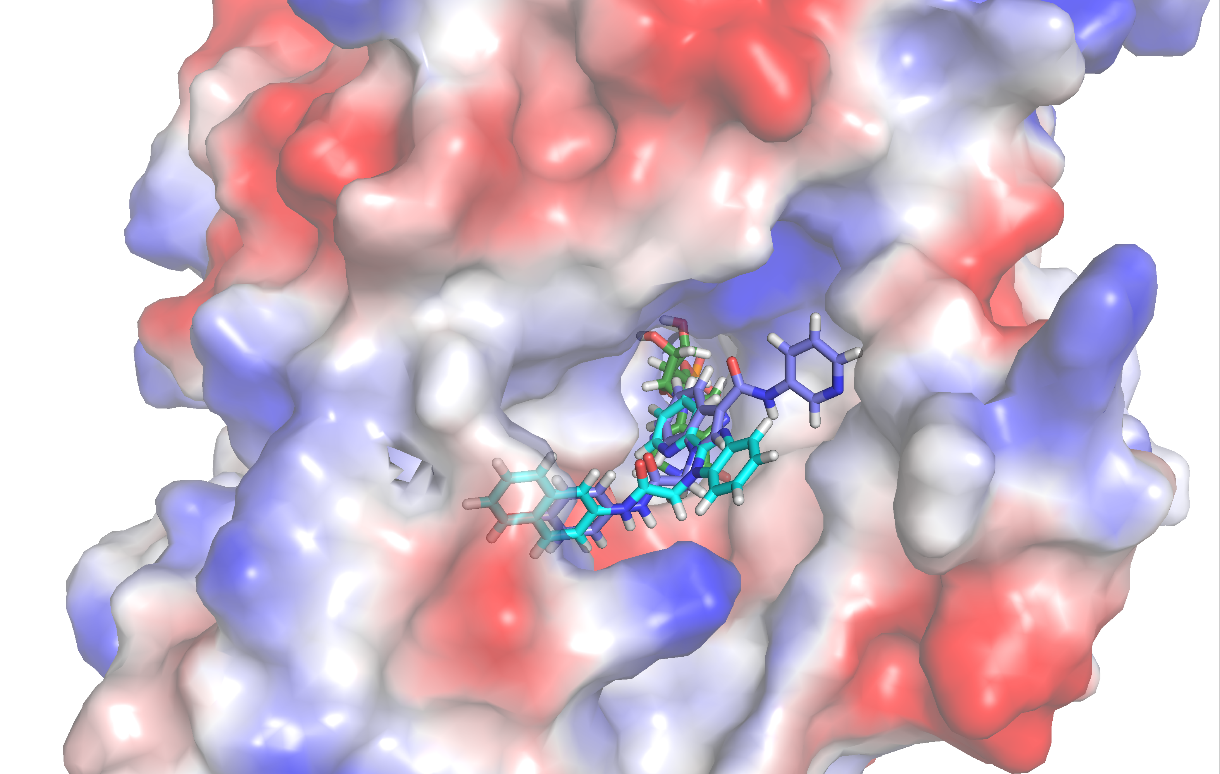


b)
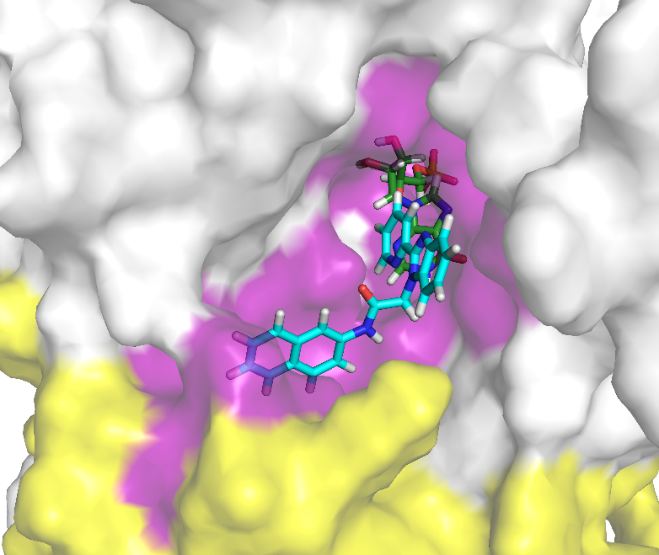
 c)
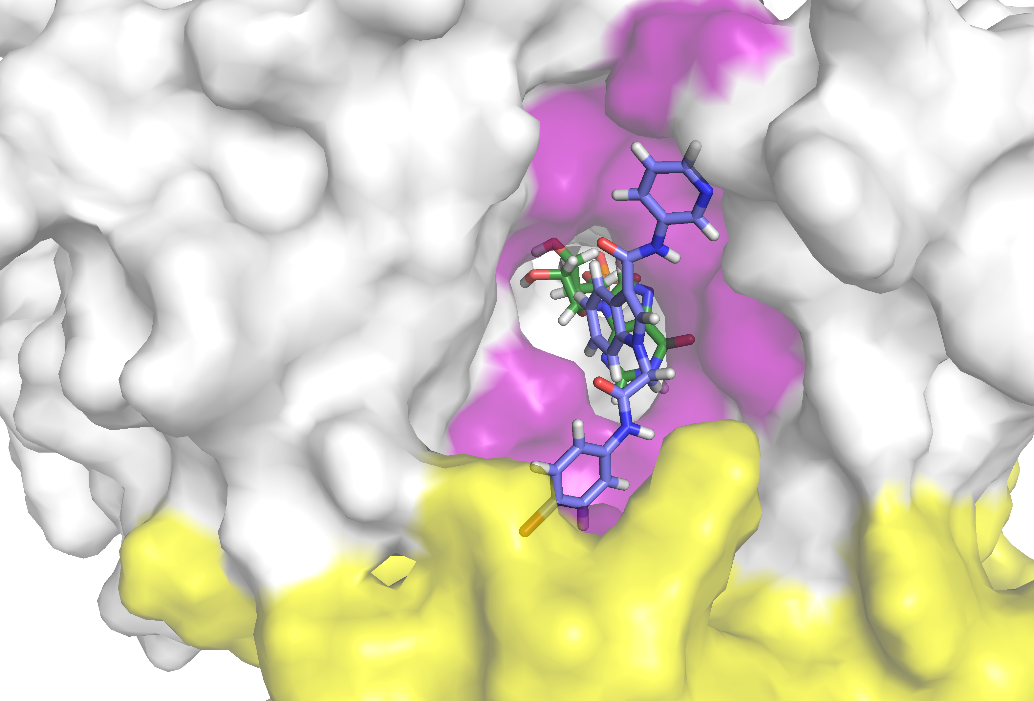


**Figure S14**. a) Binding patterns of **1** and **2** with *HpIMPDH* Mapped with electrostatic surface. b) Binding mode of **1** to *HpIMPDH c)* Binding mode of **2** to HpIMPDH (Active site is shown in purple colour, IMP shown in green colour, chain A represented in grey colour and chain D is represented in yellow colour, **1** shown in cyan colour, **2** shown in slate colour)


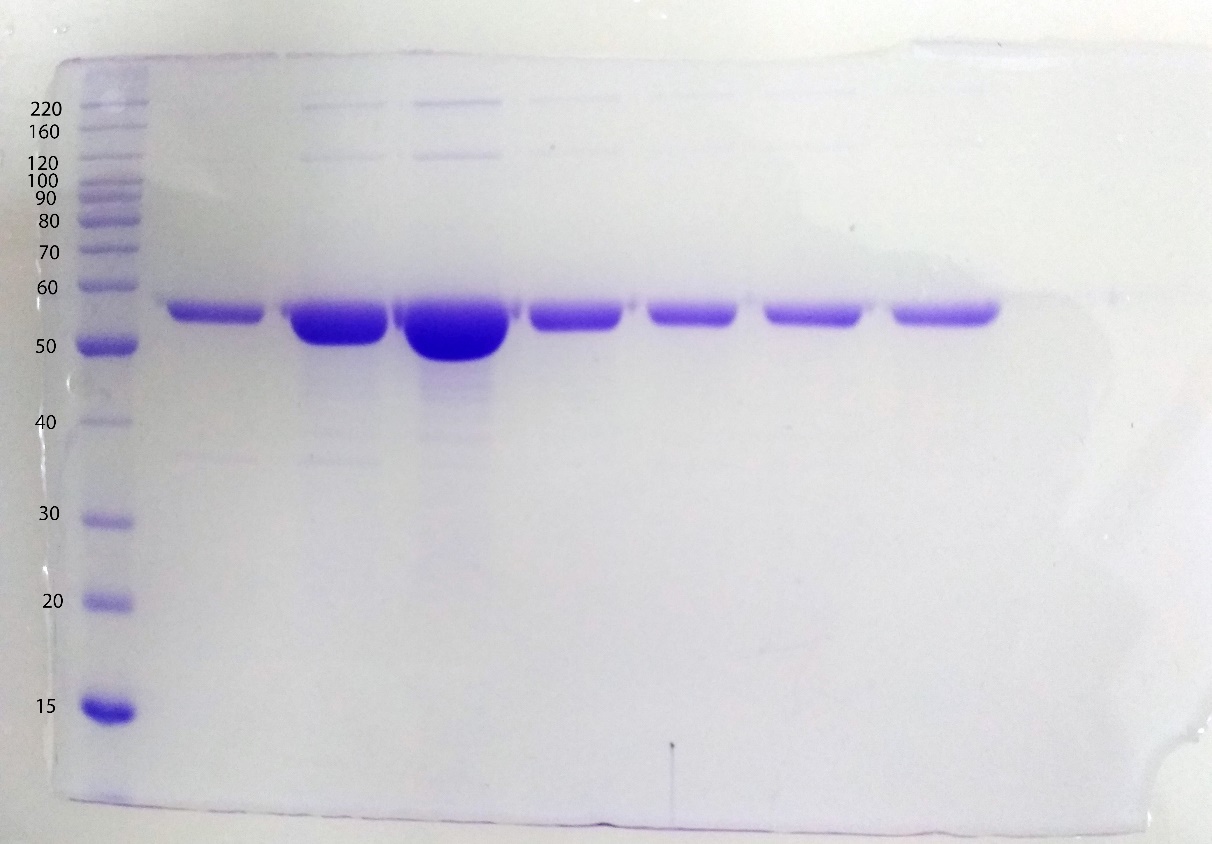


**Figure S15.** Full SDS-Page gel of purified recombinant *Hp*IMPDH


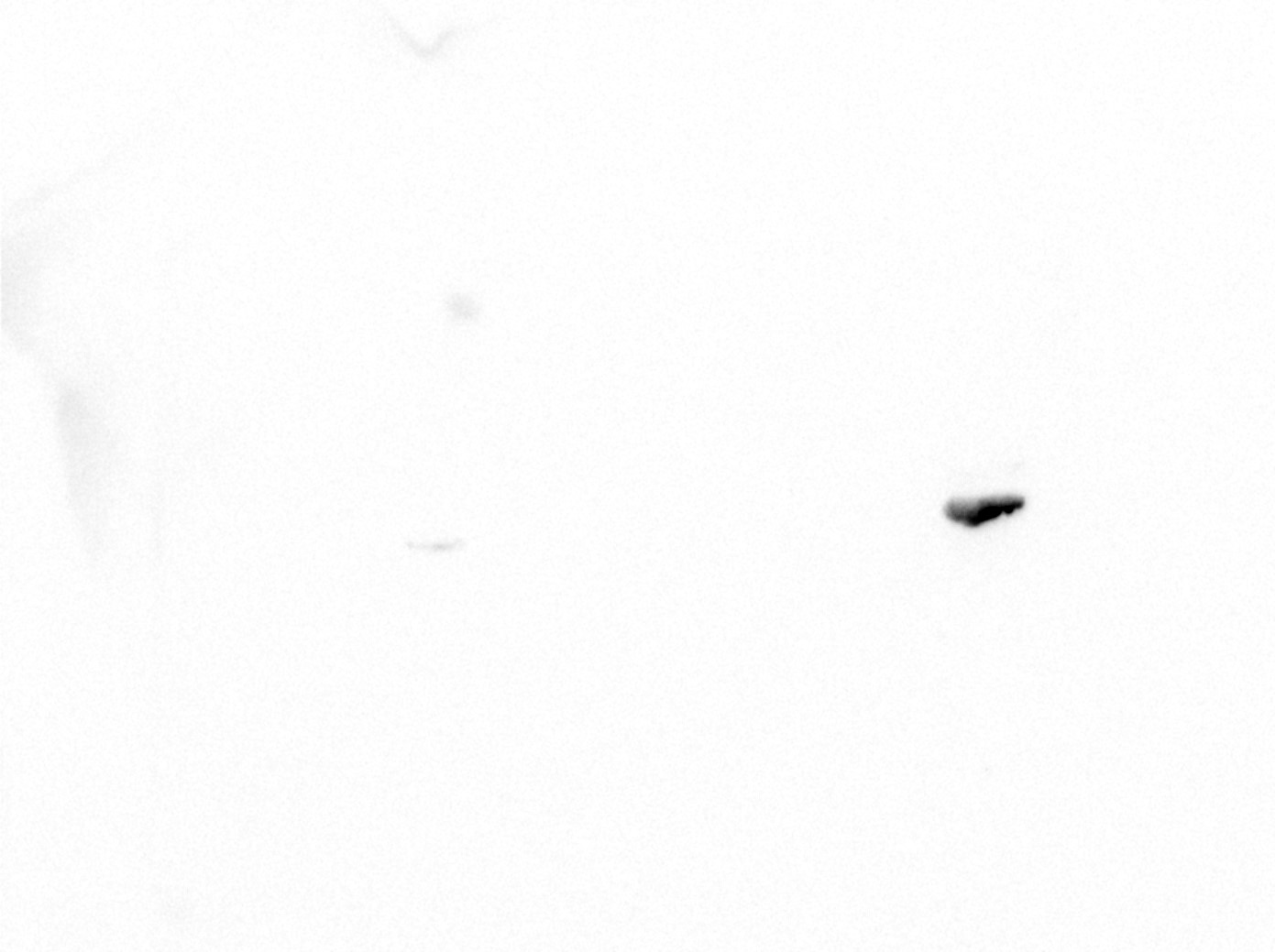


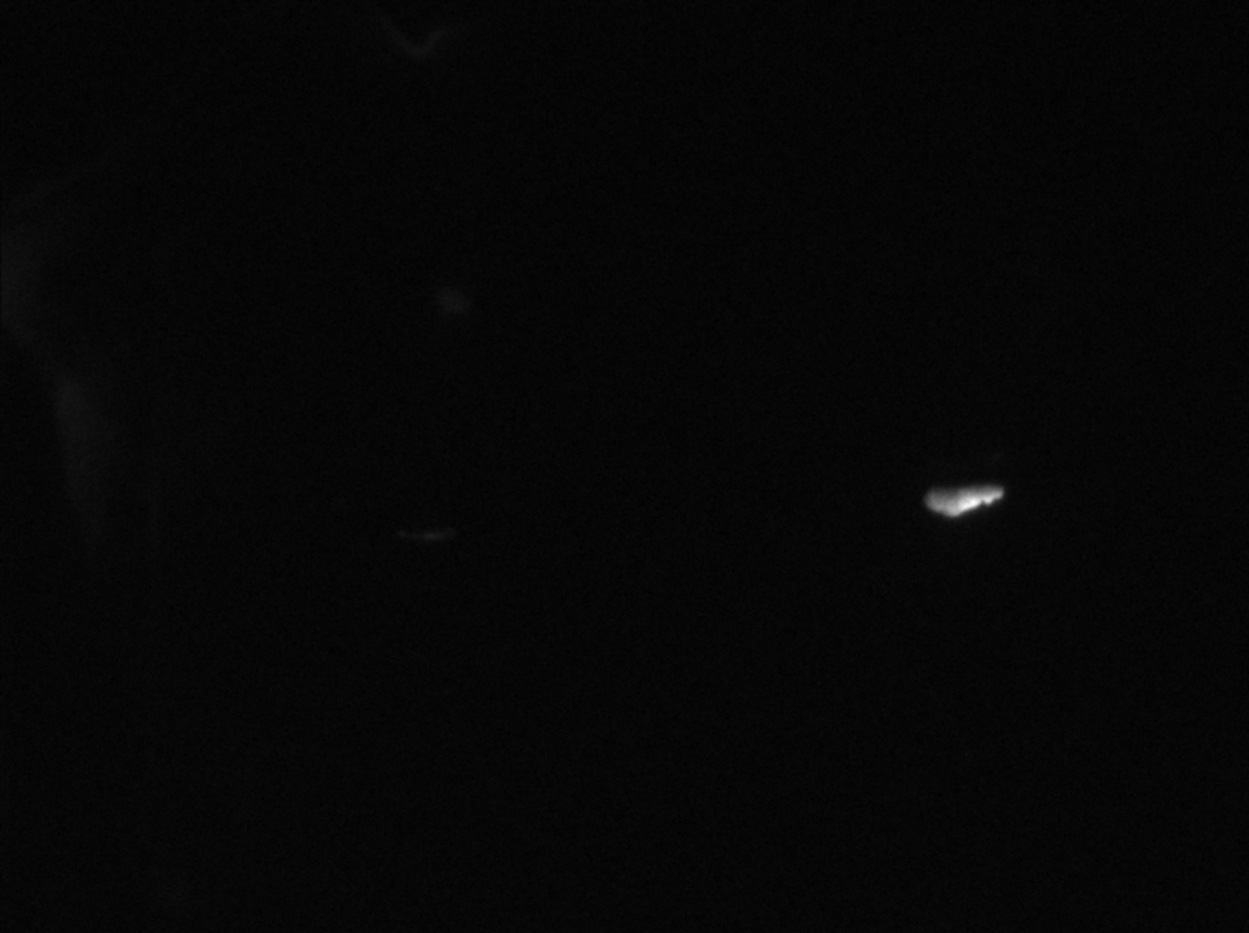


Figure S16: Western blot of purified recombinant *Hp*IMPDH at different exposures.

# References

1 Sambrook, J., Russell, D. W. & Sambrook, J. *The condensed protocols from Molecular cloning : a laboratory manual*. (Cold Spring Harbor Laboratory Press, 2006).

2 Nimmesgern, E., Fox, T., Fleming, M. A. & Thomson, J. A. Conformational changes and stabilization of inosine 5'-monophosphate dehydrogenase associated with ligand binding and inhibition by mycophenolic acid. *J Biol Chem* **271**, 19421-19427 (1996).

3 Gollapalli, D. R. *et al.* Structural determinants of inhibitor selectivity in prokaryotic IMP dehydrogenases. *Chemistry & biology* **17**, 1084-1091, doi:10.1016/j.chembiol.2010.07.014 (2010).

4 Carr, S. F., Papp, E., Wu, J. C. & Natsumeda, Y. Characterization of human type I and type II IMP dehydrogenases. *The Journal of biological chemistry* **268**, 27286-27290 (1993).

5 Buey, R. M., Ledesma-Amaro, R., Balsera, M., de Pereda, J. M. & Revuelta, J. L. Increased riboflavin production by manipulation of inosine 5'-monophosphate dehydrogenase in Ashbya gossypii. *Applied microbiology and biotechnology* **99**, 9577-9589, doi:10.1007/s00253-015-6710-2 (2015).

6 Digits, J. A. & Hedstrom, L. Kinetic mechanism of Tritrichomonas foetus inosine 5'-monophosphate dehydrogenase. *Biochemistry* **38**, 2295-2306, doi:10.1021/bi982305k (1999).

7 Labesse, G. *et al.* MgATP regulates allostery and fiber formation in IMPDHs. *Structure* **21**, 975-985, doi:10.1016/j.str.2013.03.011 (2013).

8 Umejiego, N. N., Li, C., Riera, T., Hedstrom, L. & Striepen, B. Cryptosporidium parvum IMP dehydrogenase: identification of functional, structural, and dynamic properties that can be exploited for drug design. *The Journal of biological chemistry* **279**, 40320-40327, doi:10.1074/jbc.M407121200 (2004).

9 Zhang, R. *et al.* Characteristics and crystal structure of bacterial inosine-5'-monophosphate dehydrogenase. *Biochemistry* **38**, 4691-4700, doi:10.1021/bi982858v (1999).

10 Zhou, X., Cahoon, M., Rosa, P. & Hedstrom, L. Expression, purification, and characterization of inosine 5'-monophosphate dehydrogenase from Borrelia burgdorferi. *The Journal of biological chemistry* **272**, 21977-21981 (1997).

11 Biasini, M. *et al.* SWISS-MODEL: modelling protein tertiary and quaternary structure using evolutionary information. *Nucleic Acids Research* **42**, W252-W258, doi:10.1093/nar/gku340 (2014).

12 Friesner, R. A. *et al.* Extra Precision Glide:  Docking and Scoring Incorporating a Model of Hydrophobic Enclosure for Protein−Ligand Complexes. *Journal of Medicinal Chemistry* **49**, 6177-6196, doi:10.1021/jm051256o (2006).

13 Madhavi Sastry, G., Adzhigirey, M., Day, T., Annabhimoju, R. & Sherman, W. Protein and ligand preparation: parameters, protocols, and influence on virtual screening enrichments. *Journal of Computer-Aided Molecular Design* **27**, 221-234, doi:10.1007/s10822-013-9644-8 (2013).
